# Biomimetic apposition compound eye fabricated using microfluidic-assisted 3D printing

**DOI:** 10.1038/s41467-021-26606-z

**Published:** 2021-11-09

**Authors:** Bo Dai, Liang Zhang, Chenglong Zhao, Hunter Bachman, Ryan Becker, John Mai, Ziao Jiao, Wei Li, Lulu Zheng, Xinjun Wan, Tony Jun Huang, Songlin Zhuang, Dawei Zhang

**Affiliations:** 1 Engineering Research Center of Optical Instrument and System, the Ministry of Education, Shanghai Key Laboratory of Modern Optical System, University of Shanghai for Science and Technology, Shanghai, 200093 China; 2 Department of Physics, University of Dayton, Dayton, OH 45469 USA; 3 Department of Electro-Optics and Photonics, University of Dayton, Dayton, OH 45469 USA; 4 Department of Mechanical Engineering and Materials Science, Duke University, Durham, NC 27709 USA; 5 Department of Biomedical Engineering, Duke University, Durham, NC 27709 USA; 6 Alfred E. Mann Institute for Biomedical Engineering, University of Southern California, Los Angeles, CA 90089 USA

**Keywords:** Biomimetics, Imaging and sensing, Micro-optics

## Abstract

After half a billion years of evolution, arthropods have developed sophisticated compound eyes with extraordinary visual capabilities that have inspired the development of artificial compound eyes. However, the limited 2D nature of most traditional fabrication techniques makes it challenging to directly replicate these natural systems. Here, we present a biomimetic apposition compound eye fabricated using a microfluidic-assisted 3D-printing technique. Each microlens is connected to the bottom planar surface of the eye via intracorporal, zero-crosstalk refractive-index-matched waveguides to mimic the rhabdoms of a natural eye. Full-colour wide-angle panoramic views and position tracking of a point source are realized by placing the fabricated eye directly on top of a commercial imaging sensor. As a biomimetic analogue to naturally occurring compound eyes, the eye’s full-colour 3D to 2D mapping capability has the potential to enable a wide variety of applications from improving endoscopic imaging to enhancing machine vision for facilitating human–robot interactions.

## Abstract (web summary)

Insect-like biomimetic compound eyes have many technological applications. Here, the authors present a facile fabrication scheme involving microfluidics assisted 3D printing that permits to completely separate design, optimization and construction of optical and sensor components.

## Introduction

Survival of the fittest has continuously driven the evolution and improvement of compound eyes^1,2^. Even early examples of arthropods dating back to the Cambrian era had evolved faceted compound eyes^3,4^ which enabled them to perceive their environment based on visual phototaxis^5^. A compound eye consists of a group of ommatidia which are oriented in different directions to provide arthropods with panoramic vision accompanied with other useful visual advantages including depth perception, low-aberration detection of their surroundings, and high-sensitivity motion tracking. Each ommatidium includes a corneal facet lens for light collection, a crystalline cone and a rhabdom for light transmission, and pigment cells for optical isolation to minimize crosstalk^6,7^. The resulting outstanding visual performance enabled by this compound design has been widely exploited for a diverse range of applications such as endoscopic examination^8^, robot navigation^9,10^ and surveillance^11^. Natural compound eyes have inspired various optical systems with artificial microlens arrays^12–19^, and a significant amount of research has been devoted to improving the fabrication and design of these systems.

Most artificial compound eyes that have been previously demonstrated rely on conventional microfabrication techniques. For example, some compound eyes are fabricated by transferring planar microlens arrays (or moulds) to the surface of a hemisphere^20–22^. Although these planar microlens arrays are relatively easy to fabricate using thermal reflow^23,24^, laser-induced forward transfer^25,26^, laser ablation^27^, jet printing^28,29^ or microfluidic manipulation^30–32^ techniques, transferring the pattern to a spherical surface can affect the uniformity of the lens and thus the performance of the system. Even though issues related to 3D fabrication can be resolved by applying advanced microfabrication techniques, such as 3D laser writing, laser lithography, chemical etching, or two-photon polymerization^33–42^, a fundamental problem still exists. Namely, the images produced from existing 3D artificial compound eyes do not match with current commercial planar imaging sensor technology. Automatic matching of the image from a compound eye to a planar imaging sensor can significantly reduce the complexity of image-processing algorithms and also will reduce the number of sensors required for the system. Deformable optoelectronics, in which an array of photodetectors are curved to match to a compound eye^43–46^, provide a potential solution for the aforementioned matching problem; however, the deformable curvature might be a key factor that would limit the size of the compound eye.

In order to overcome all these shortcomings, we have developed a biomimetic apposition compound eye (BAC-eye) using a unique fabrication method that combines 3D printing with microfluidic-assisted moulding to pattern 522 microlenses, in an omnidirectional manner, across the surface of a hemisphere. Each microlens of the BAC-eye is optically connected to the flat base of the hemisphere with an optical pipe that consists of a refractive index-matched waveguide. The flat base of the compound lens can be directly attached to any planar image sensor to enable full-colour, wide-field-of-view imaging. This effectively makes the BAC-eye an accurate recreation of a natural compound eye, yielding a compact form factor (5 mm in diameter vs. 4 mm for the compound eyes of a dragonfly) and a large viewing angle (170° vs. 150° to 180° in most natural compound eyes). As a proof-of-concept demonstration, we captured full-colour, wide-angle panoramic images, and demonstrated accurate position tracking of a point source. The unique fabrication method presented herein enables the fabrication of highly adaptable biomimetic compound eyes that are compatible with any existing planar imaging sensors and greatly simplifies the optics and electronics required for obtaining a digital 3D panoramic view. With its unique 3D to 2D mapping capability, the 3D BAC-eye presented here opens up many applications in photonics, sensing, and imaging.

## Results

### Fabrication of the 3D BAC-eye

**Fig. 1:**
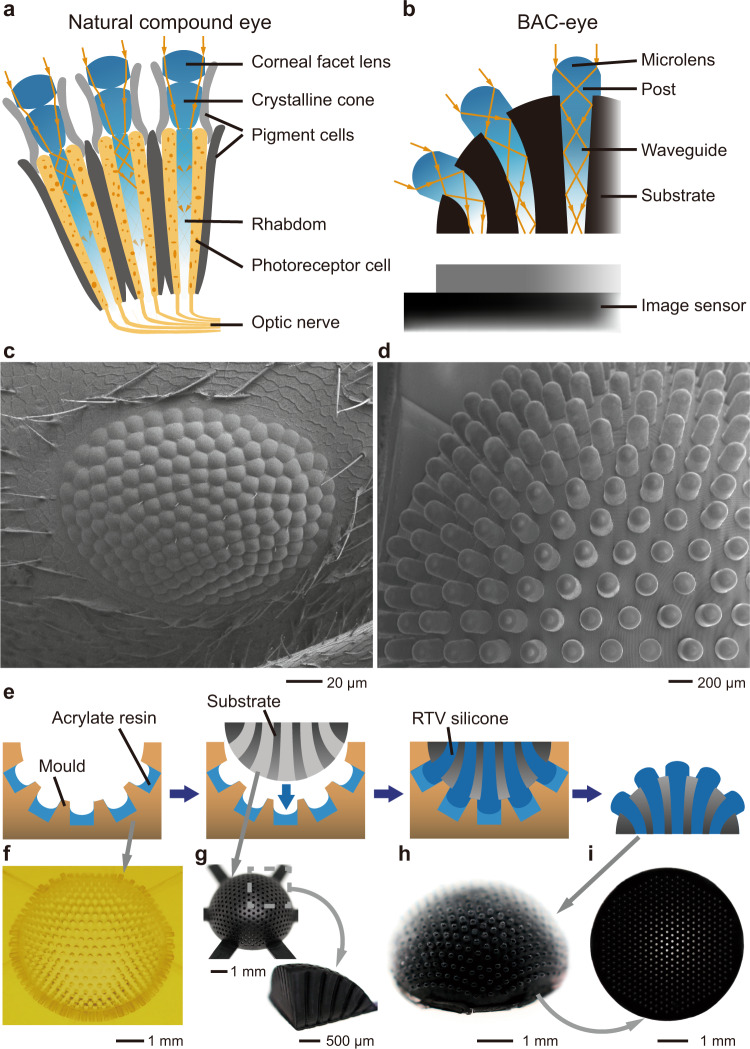
Illustrations of the fabrication procedure and images of the BAC-eye. **a** Anatomical structure of an arthropod compound eye. **b** Labelled cross section of a BAC-eye. **c** SEM image of a compound eye of the Asian needle ant, *Brachyponera chinensis*. **d** SEM image of a BAC-eye. **e** Illustration of the main steps of the fabrication procedure. The BAC-eye is produced in a hemispherical substrate by casting it in a prepared mould. **f** Image of the 3D-printed mould. **g** The 3D-printed substrate and a quarter sectional slice of the substrate. **h** Image of a BAC-eye after release from the mould. **i** A view showing the flat bottom of the BAC-eye.

The design of the 3D BAC-eye follows the anatomical structure of an apposition compound eye (Fig. 1a). Each microlens on the BAC-eye has the same function as the corneal facet lens of a natural eye. The cylindrical post and the silicone-elastomer waveguide function as a crystalline cone and a rhabdom, respectively (Fig. 1b). The internal structure of the artificial eye mimics the function of pigment cells to reduce optical crosstalk. The number of ommatidia in the BAC-eye (522) is comparable with that of bark beetles (*Dendroctonus rufipennis*, average count: 272; *Dendroctonus valens*, average count: 372), ants (*Temnothorax albipennis*, average count: 300 (male) and 171 (queens); *Brachyponera chinensis*, average count: 168 (worker) (Fig. 1c)), and fruit flies (*Drosophila melanogaster*, average count: 730)^47–49^. Figure 1d shows a top-view SEM image of the BAC-eye. It has a radius of 2.5 mm and its microlenses are hexagonally and omnidirectionally distributed across the hemispherical dome. The most peripheral ommatidia are oriented at ±85° with respect to the vertical axis, extending the viewing angle of the BAC-eye to 170°.

The fabrication process and the components used in the fabrication are illustrated in Fig. 1e–i (additional details provided in Supplementary Figs. 1–4). First, a mould with an open hemispherical pit is 3D-printed using a projection micro-stereolithography 3D printer^50,51^. The surface of the hemispherical mould is patterned with 522 cylindrical microholes, each with a diameter of 180 μm, that are arranged omnidirectionally along the surface of the hemisphere (Fig. 1f and Supplementary Fig. 2). The process of forming a convex lens mould within these cylindrical holes, however, requires precision handling; due to the small size of the microholes and current limitations in the resolution of 3D-printing technology, the curvature cannot be encoded into the mould directly. Therefore, a microfluidic-assisted moulding technique, which leverages surface tension, was used to form a proper concave shape within each microcavity.

**Fig. 2:**
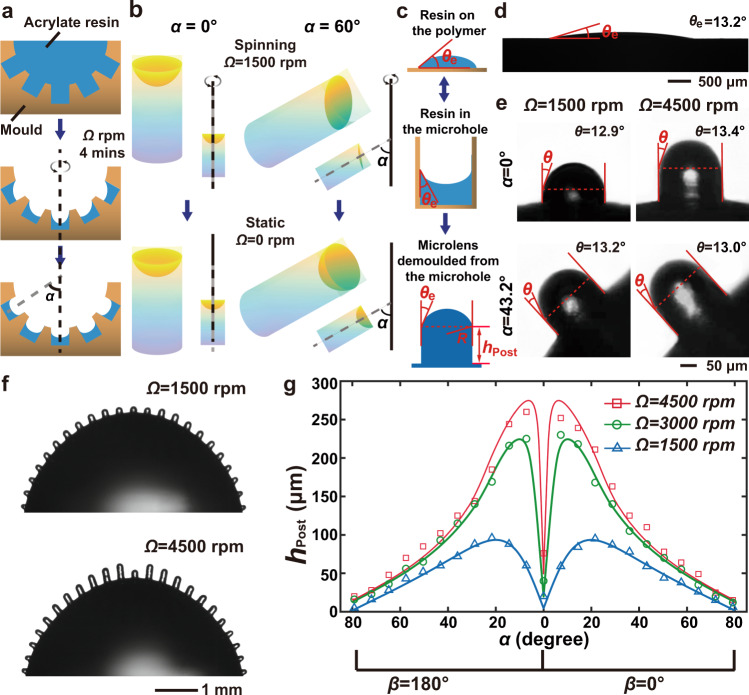
Design of the mould and morphologic characterization of the BAC-eye. **a** Illustration showing the fabrication of the mould. **b** The surface profiles of the acrylate resin in the microholes in different orientations during dynamic (while spinning) and static (after spinning) equilibrium. **c** A comparison between the surface profiles of the acrylate resin on the (top) flat polymer substrate, (middle) in the microhole, and (bottom) after the microlens is demoulded from the microhole. **d** Contact angle measurements for the acrylate resin on the photosensitive polymer. **e** Microscope images of the artificial ommatidia. The microlenses in the different orientations and as produced under different spin rates have a uniform curvature. **f** Images of a single row of the ommatidia along the curved surface of the BAC-eye. Different heights are produced based on the location of the posts and the balance of forces during the fabrication process. **g** Experimentally measured (data point markers) and simulation results (solid curves) of the post height distribution across the surface of the hemisphere, with respect to rotational rate.

The procedure of the microfluidic-assisted moulding is illustrated in Fig. 2a, Supplementary Fig. 1, and Supplementary Note 1. To form the microlens mould, a hemispherical pit with cylindrical microholes is first filled with acrylate resin. The mould is then spun around its central axis at a spin rate of *Ω* rpm for 4 min. As the mould is spun, a portion of the acrylate resin is ejected from the microholes due to the centrifugal force generated by the spinning process. The amount of resin that remains within each hole is a function of the spin parameters and the location of the hole within the mould. Figure 2b and Supplementary Fig. 5 presents results from numerical simulations that were performed to study the surface profile of the acrylate resin in the microholes at different orientations (polar angle, *α*, and azimuthal angle, *β*, as defined in Supplementary Fig. 3) before and after spinning the mould. While spinning, the surface of the liquid-state acrylate resin in the on-axis microhole (*α* = 0°) becomes a symmetrical parabolic shape, while the resin in the off-axis microholes (*α* ≠ 0°) gradually inclines towards the outer side of the microhole as the angle *α* increases. The amount of acrylate resin remaining in the microhole decreases as the spinning speed increases (Supplementary Fig. 5). Moreover, in the off-axis microholes (*α* ≠ 0°) close to the centre, most of the acrylate resin ascends up the side of the microhole and spills out (Supplementary Fig. 6). In contrast, the tilted microholes on the edge of the hemispherical pit hold more acrylate resin.

When the spinning stops, the surface tension dominates and deforms the surface of the acrylate resin into a concave shape within all the microholes. More specifically, the radius of curvature of the concave surface can be described by the contact angle, *θ*_e_, between the three phases under thermodynamic equilibrium, i.e., $R=d/(2\,\cos ({\theta }_{{{{{{\rm{e}}}}}}}))$ (Fig. 2c). The equilibrium contact angle, *θ*_e_, is 13.2°, as measured in Fig. 2d for the system used in these experiments. With a uniform curvature achieved within each microhole, the liquid-state acrylate resin within each chamber is then UV cured for 15 min. The convex surface of each ommatidium can be obtained as the complimentary mould of the acrylate resin in the microholes using microfluidic-assisted moulding. To analyse this performance of this replication process, two moulds, each of which consisted of a single row of microholes on the bottom of the hemispherical pit, were prepared by spinning the acrylate resin with speeds of 1500 and 4500 rpm, respectively. Figure 2e shows side-view optical images of the replicated microlenses at different orientations and spinning speeds. The profiles of the microlenses are nearly identical, demonstrating that their shape is independent of their orientation and the rotational speed, which is consistent with the assumption that the surface tension dominates the formation of the concave lenses. The radius of curvature of each microlens is 91.9 ± 0.8 μm (Supplementary Fig. 7), which is in good agreement with the theoretical prediction, *R* = 92 μm (Supplementary Equation 9 in Supplementary Note 2). As expected, the height of the cylindrical post (*h*_Post_) depends on both the rotational speed (*Ω*) and the orientation of the microholes (*α*) in the mould as shown in Fig. 2f. This is because a larger spin speed removes more of the acrylate resin and the microhole close to the centre holds less acrylate resin, resulting in a deeper microhole, and subsequently higher complementary posts. Figure 2g shows the height of the post as a function of *α* at different rotational speeds *Ω*. The experimental data (markers) agrees well with the calculations (solid curves). Even though the height of each post is different for each ommatidium in the BAC-eye, the curvature of the microlens on each post is the same. This means that all the ommatidia have an identical relative aperture.

After the concave lens mould surfaces are fabricated, a hemisphere that is complementary to the patterned mould is 3D-printed using a UV curable diacrylate polymer (refractive index *n*_Polymer_ = 1.46) that is mixed with Sudan Black 3 solvent dye (Fig. 1g). The hemisphere consists of 522 hollow pipelines, or tapered channels, which connect the hemispherical surface to the flat base. Additional details about the design and the 3D model of this complementary substrate can be found in Supplementary Note 3, as illustrated in Supplementary Fig. 3 and Supplementary Fig. 4. The hemisphere is then inserted into the mould and the hollow pipelines in the substrate are aligned with the cylindrical microholes in the mould. The empty pipelines and the concave microholes are filled with silicone by immersing the combined system in a room-temperature-vulcanizing (RTV) silicone in a liquid state. After curing for 4 h, the RTV silicone solidifies into an elastomer. Separating the hemisphere from the mould yields a complete BAC-eye, as shown in Fig. 1h. Each ommatidium consists of a microlens with a radius of 90 μm capped on a cylindrical polymer post. These ommatidia are optically connected to the bottom of the BAC-eye through the pathway formed by the silicone-elastomer waveguide (refractive index *n*_Silicone_ = 1.50) which was formed in the hollow pipeline. The diameters of the silicone-elastomer waveguides gradually narrow down from the ommatidia (*d*_T_ = 157 μm) to the bottom of the BAC-eye (*d*_B_ = 100 μm). This design serves to increase the separation between individual sources. The outputs of the waveguides are hexagonally arranged at the flat base of the BAC-eye (Fig. 1i). Since the bottom of the BAC-eye is physically flat and the 3D array of the surface microlenses has been mapped to a regular hexagonal 2D array via these waveguides, this system can be directly matched to any commercial planar image sensor.

### Optical characterization of the BAC-eye

In order to ensure that the BAC-eye maintains a high optical fidelity comparable to a natural compound eye, we optimized the performance of the waveguides and hemispherical substrate of the device. The hemispherical element, which serves as the supporting body of the BAC-eye, is comprised of a photosensitive polymer dyed with Sudan Black 3 solvent dye. The optical waveguides that connect the cylindrical posts of the artificial ommatidia and the bottom surface of the BAC-eye are patterned within this photosensitive polymer. The dye is used to absorb any stray light that escapes from the waveguides and hence acts to eliminate optical crosstalk between adjacent waveguides. Details about the optical density of the photosensitive polymer dyed with Sudan Black 3 solvent dye are discussed in Supplementary Note 4. When the concentration is 1500 μg/mL, the photosensitive polymer with thickness of 9.8 μm has optical density of 3 over the entire visible spectrum (Supplementary Figs. 8 and 9). The RTV silicone used for ommatidia and waveguides is transparent in the range of 400–1100 nm (Supplementary Fig. 10).

**Fig. 3:**
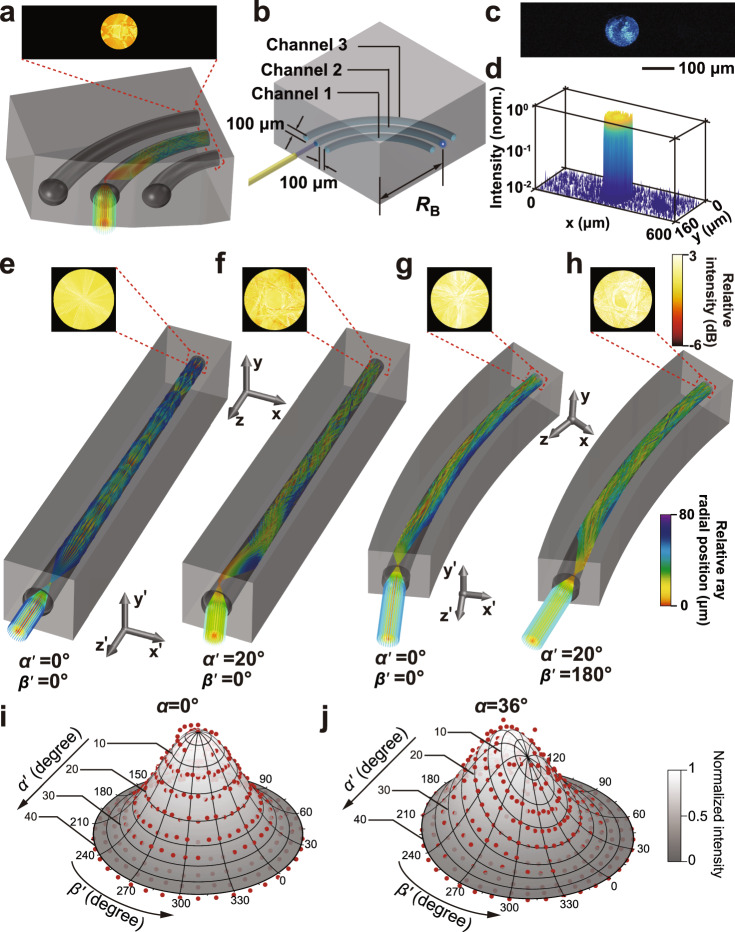
Optical performance of the artificial ommatidia. **a** Simulation of optical crosstalk among the three ommatidia with orientations of (*α* = 64.8°*, β* = 0°), (*α* = 72°*, β* = 0°), and (*α* = 79.2°*, β* = 0°) and intensity distributions at the proximal ends of the waveguides when the light is incident on the middle ommatidium. **b** A three-channel model to measure the crosstalk among the curved silicone waveguides. **c** Image captured at the output panel showing the light intensity at the proximal ends of the three silicone waveguides. **d** The measured light distribution at the output panel. **e**–**h** Simulation of ray tracing in the ommatidia with orientations of (**e**, **f**) (*α* = 0°*, β* = 0°) and (**g**, **h**) (*α* = 36°*, β* = 0°) and light intensity distributions at the proximal ends of the waveguides when the light is incident from different angles. **i**, **j** The angular sensitivity function of the ommatidia with orientations of (*α* = 0°*, β* = 0°) and (*α* = 36°*, β* = 0°), respectively. Red dots: the normalized intensity obtained from experimental measurements. Grey surface: Gaussian-fitting surface.

To further test the transmission properties and optical crosstalk between the waveguides of each ommatidium of the BAC-eye, simulation models of three ommatidia were established based on the actual structure and physical properties of the ommatidium as shown in Fig. 3a, Supplementary Figs. 11 and 12. The collimated light was incident on the microlens of a specific ommatidium. We could observe the light output only from the end of the corresponding waveguide. Moreover, three curved silicone waveguides were fabricated within the photosensitive polymer mixed with 1500 μg/mL solvent dye, as shown in Fig. 3b. The diameter of each silicone waveguide and the separation distance between each optical pathway are each 100 μm, consistent with the dimensions of the BAC-eye. The bend radius and angle of the waveguides are 600 μm and 90°, respectively. A multi-mode fibre with a 450 nm light source was connected to the middle waveguide (Channel 2). Figure 3c shows an image from the outputs of the three waveguides when only Channel 2 is illuminated. We could not visibly detect any light from Channel 1 or Channel 3, which was consistent with the light distribution measured from the proximal ends of the waveguides (Fig. 3d). The extinction ratios between Channel 2 and Channel 1 and between Channel 2 and Channel 3 are 16.1 and 15.2 dB, respectively. These results are consistent with the optical density measurements from Supplementary Fig. 8, and confirm that the photosensitive polymer mixed with the solvent dye can eliminate any optical crosstalk between the waveguides.

We also analysed the coupling and propagation of light within each ommatidium of the BAC-eye using a ray tracing method. In this simulation, collimated light, which is incident on the microlens of the ommatidium with an incident angle (polar angle *α*′ and azimuthal angle *β*′, as defined in Supplementary Fig. 13), is coupled into the waveguide. We performed these simulations with a straight waveguide and two curved waveguides with orientations of (*α* = 36°*, β* = 0°) and (*α* = 79.2°*, β* = 0°), respectively, to mimic the curvature of the optical pathways within the BAC-eye (Fig. 3e–h and Supplementary Figs. 14–16). Since the refractive index of the substrate is lower than that of the waveguide, total internal reflection ensures the propagation of the light inside the waveguide. Owing to the oblique incidence and the non-axisymmetric multiple reflection inside the curved waveguide, the distribution of the rays deviates from the centre at the proximal end of the waveguide. In spite of that, the simulation confirms that the light can be well confined in the ommatidia and efficiently transmit to the base of the eye. In addition, based on the experimental measurement, we found that the optical loss, including the coupling loss into/out of the ommatidium and the transmission loss, is 5.37 dB (details about the measurement of optical loss can be found in Supplementary Note 5 and Supplementary Fig. 17). The loss is attributed to the bending and narrowing of the waveguide.

In addition to the light propagation inside the ommatidia, the 3D nature of this system means that light enters each ommatidium at different angles; therefore, the angular sensitivity of the ommatidia was investigated. In the simulation, the light intensity distribution at the proximal ends of the waveguide was analysed and transmittance, i.e., the ratio between the integral of the output intensity at the proximal end and the incident intensity on the microlens, was calculated (Supplementary Figs. 18 and 19). Slight bending loss was observed in the highly-curved waveguides. It is worth noting that the substrate can absorb the leakage of the light and the optical crosstalk between adjacent optical pathways can be efficiently avoided. Furthermore, the angular sensitivity function of three ommatidia at three different orientations of (*α* = 0°*, β* = 0°), (*α* = 36°*, β* = 0°), and (*α* *=* 79.2°, *β* = 0°) was experimentally measured, respectively. Supplementary Fig. 20 schematically shows this experiment setup, where a collimated light beam illuminates the surface of the BAC-eye, and the angular sensitivity function of each ommatidium (*α* = 0°*, β* = 0°), (*α* = 36°*, β* = 0°) and (*α* = 79.2°*, β* = 0°) is obtained by measuring the transmitted light intensity from each ommatidium as a function of the incident angle of the collimated light beam (details about the experimental measurement and the simulation of angular sensitivity can be found in Supplementary Note 6). The incident collimated light beam can be rotated around the BAC-eye at any angle (*α*′ or *β*′) as defined in Supplementary Fig. 20. Figure 3i and j and Supplementary Fig. 21 show the angular sensitivity function of the three ommatidia with the orientations of (*α* = 0°*, β* = 0°), (*α* = 36°, *β* = 0°), and (*α* = 79.2°, *β* = 0°). The light intensity is normalized to the maximum value measured at the central ommatidium (*α* = 0°). The plotted red points were obtained from experimental data, while the surface is a Gaussian fit of the experimental data. The ommatidia with the orientations of (*α* = 0°, *β* = 0°), (*α* = 36°, *β* = 0°), and (*α* = 79.2°, *β* = 0°) have the highest intensity at incident angles of (*α*′ = 0°, *β*′ = 0°), (*α*′ = 12°, *β*′ = 180°), and (*α*′ = 30°, *β*′ = 180°), respectively. The acceptance angle of each ommatidium, which is defined as the full width at half maximum of the angular sensitivity function, is about 44°. The wide acceptance angle is attributed to the large diameter of the waveguide, where a large number of propagating modes are allowed^52^. These experiments suggest that light collected by each ommatidium is efficiently transmitted to the bottom surface of the BAC-eye and can be directly detected by a planar image sensor regardless of the incident angle relative to the artificial eye.

### Panoramic imaging using the BAC-eye

**Fig. 4:**
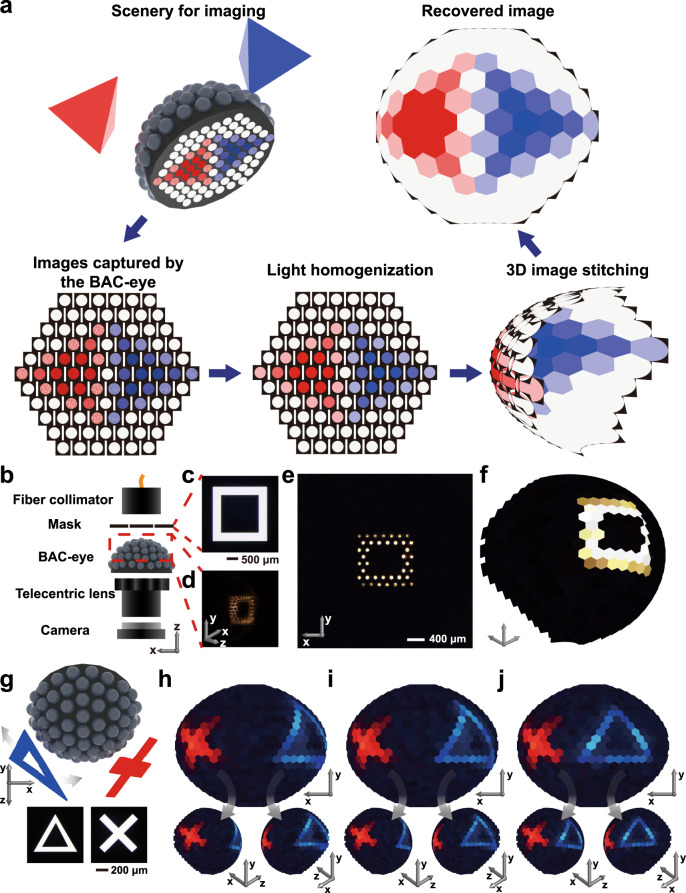
Imaging using the BAC-eye. **a** Workflow for image acquisition and processing by the BAC-eye. The colour scale represents the intensity level of the detected signals. **b** Schematic diagram of the experimental setup of the imaging system. **c** The square mask used for image detection experiments. **d** The illuminated square pattern as detected by the BAC-eye. The image of the illuminated pattern on the artificial ommatidia is captured by a single-lens reflex (SLR) camera equipped with a macro lens. **e** The square pattern image captured from the bottom of BAC-eye. **f** The digitally reconstructed hemispherical image. **g** Illustration of the hemispherical imaging for a red cross pattern whose centre is fixed at an angular position of (*α* = 60°, *β* = 0°) and a blue triangular pattern moving from the side (*α* = 85°, *β* = 180°) toward the red cross. **h**–**j** The reconstructed images showing the triangle as it travels from (*α* = 60°, *β* = 180°), (*α* = 40°, *β* = 180°), and (*α* = 20°, *β* = 180°). The digitally generated callouts provide stereoscopic vision (for a human observer) of the hemispherical images.

In contrast to conventional macro imaging lenses, the BAC-eye is capable of forming wide-angle panoramic images. Figure 4a shows the working principle for capturing panoramic images by coupling a BAC-eye to a planar complementary metal–oxide–semiconductor (CMOS) camera. Light emitted or reflected from an object, such as the red or blue regular tetrahedron in Fig. 4a, is captured by each ommatidium and guided to the bottom of the BAC-eye where its image is recorded by a colour camera. The sub-image on the camera that corresponds to the light from each ommatidium is then homogenized by taking the average value of the light from each ommatidium. This averaging is needed because each ommatidium projects its light across ~80 × 80 pixels of the planar imaging sensor. Finally, a panoramic image of the object is generated on a hemisphere by digitally stitching the images from each ommatidium together while accounting for the orientation of each ommatidium on the outer surface of the BAC-eye (details in ‘Methods’ and Supplementary Fig. 22). The resolution of the BAC-eye is dependent upon the total number of ommatidia.

Figure 4b shows the panoramic imaging of a square as visualized through the BAC-eye. Details of the panoramic imaging system are given in ‘Methods’. A mask with a square object which is 300 μm in width (Fig. 4c) was placed in front of the BAC-eye to project a square onto the BAC-eye. Figure 4d shows a side-view optical image of the projected light on the BAC-eye, as taken using another digital single-lens reflex camera equipped with a macro lens at an angle of *α* = 15° and *β* = 180°. A telecentric lens was used to magnify the image at the flat base of the BAC-eye and project it onto the CMOS camera. The telecentric lens is not necessary in practical applications, and the BAC-eye can be directly attached to an image sensor; the telecentric lens was used in this experiment solely to magnify the image and improve spatial sampling. Figure 4e and Supplementary Fig. 23a show the image of the square on the camera. The corresponding panoramic 3D image of the square slot is shown in Fig. 4f.

We also demonstrated that the BAC-eye can image objects at different angular positions with a visible angle ranging of 170°. Figure 4g schematically shows the experimental setup for imaging of two objects at different angular positions. A red cross with a line width of 300 μm was placed at a fixed angular position of *α* = 60° and *β* = 0° (the centre position of the cross), while a blue triangle with a line width of 200 μm was moved from the side toward the red cross. The corresponding panoramic views for the centre position of the blue triangle at (*α* = 60°, *β* = 180°) (Fig. 4h), (*α* = 40°, *β* = 180°) (Fig. 4i), and (*α* = 20°, *β* = 180°) (Fig. 4j) are reconstructed. The patterns are clearly recognized and the triangle at different angular positions is imaged with a high uniformity in size and shape. The image detected by the CMOS camera for the triangle centred at an angular position of *α* = 20° and *β* = 180° is illustrated in Supplementary Fig. 23b. In this demonstration, since coherent monochromatic lasers were used as the illumination sources, interference of the different portions of the incident light occurred; therefore, granular speckle patterns can be observed in the sub-images of the ommatidia. In nature, arthropods can quickly detect and escape from predators and track prey, all based on the information, e.g., position, direction and speed of motion, provided by their peripheral vision. The advantages of wide-angled, motion-based sensing in applications range from macro surveillance functions to navigational functions in endoscopic surgeries.

### 3D point-source tracking with the BAC-eye

**Fig. 5:**
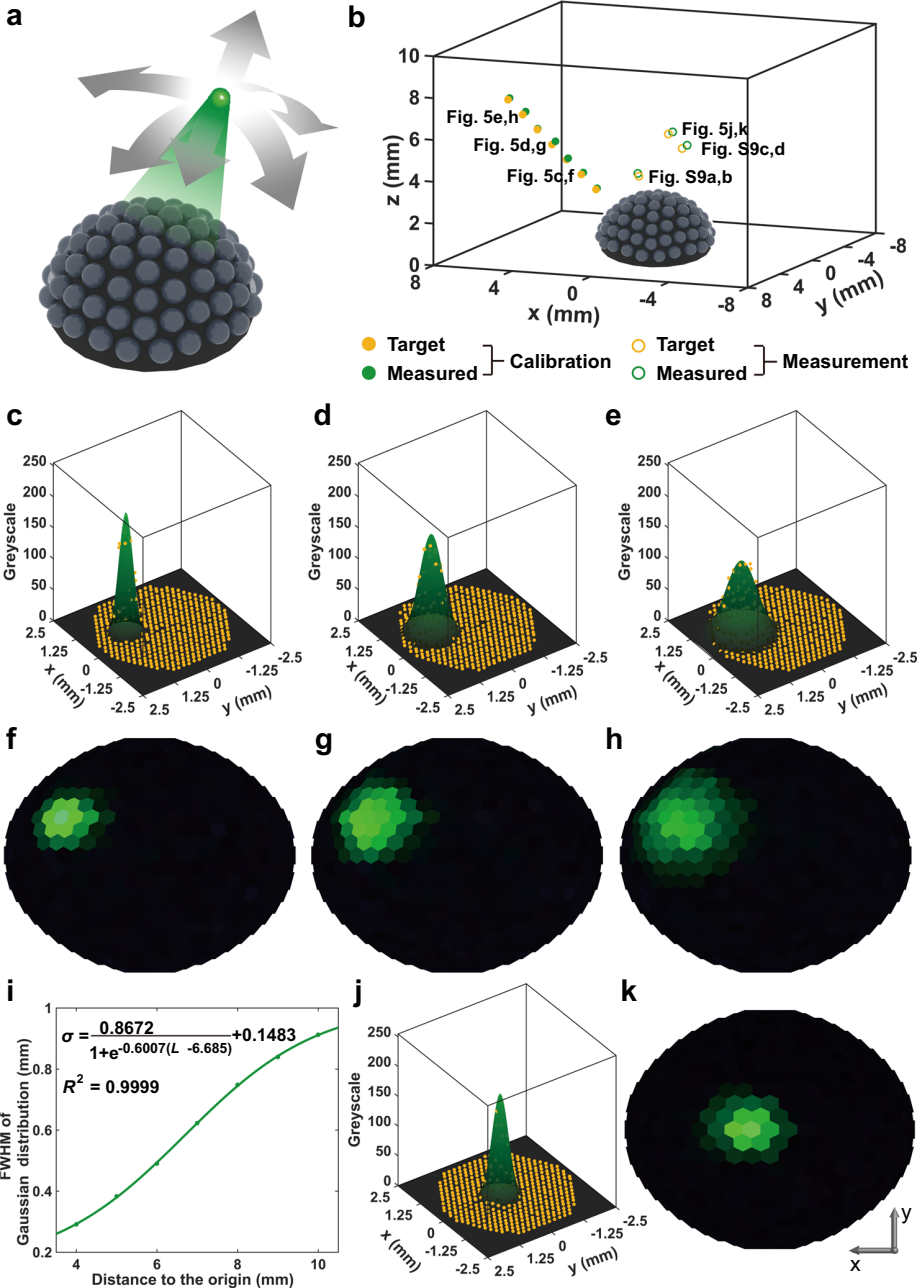
Tracking the position of a light point source using the BAC-eye. **a** Schematic diagram of the light point source tracking experiments. **b** The positions of the light spots. The yellow and green solid dots are the target and measured positions used for calibration, respectively. The yellow and green circles are the target and measured positions from experiments where the light location is not known a priori. **c**–**e** The light distribution collected by the BAC-eye and **f**–**h** the corresponding hemispherical images of the light spots at a radial distance 5, 7, and 9 mm away from the original point. Yellow dots are the average grayscale measured from the proximal ends of the waveguides. The colour scale represents the intensity level of the detected light. **i** The relation between the full width at half maximum (FWHM) of the light distribution obtained by the BAC-eye and the distance from the original point to the light spot. **j** The light distribution obtained for the light spot at a nominally-unknown position and **k** the corresponding hemispherical images of the light spot.

The natural compound eyes of fruit flies and worker bees have poor resolution with respect to static images, but they are highly sensitive to 3D motion detection. Similarly, the BAC-eye can be used to track the position of objects in three dimensions. In contrast to conventional monocular vision systems, compound eyes have the visual advantage of depth perception. Supplementary Fig. 24 illustrates a scenario where objects at different distances are detected using imaging systems equipped with a conventional fisheye lens and the BAC-eye, respectively. The images captured by the fisheye lens are identical, indicating that it cannot distinguish between the absolute distances of the objects. In contrast, it is feasible for the BAC-eye to determine the object distance. In this section, we demonstrate the 3D position tracking of a green light point source with a BAC-eye, as shown in Fig. 5a. The diverging green light emitted from an optical fibre is captured by a BAC-eye and projected onto a CMOS camera. The light spots projected onto the CMOS camera through the BAC-eye (Fig. 5c–h) change in position and diameter depending on the angular position of the point source and the distance between the point source and the centre point of the BAC-eye. The light spot on the CMOS camera can be fitted neatly with a Gaussian function (Fig. 5c–e). Figure 5f–h shows the corresponding panoramic views of the point source at three distances. When the point light source moves away from the BAC-eye, the illumination area incident on the compound eye becomes large and the diameter of the light spot image on the camera increases, and vice versa (Supplementary Fig. 25). Therefore, the centre position and the width of the imaged light spot on the CMOS camera can be calibrated to obtain the 3D position of the point source as it moves. Figure 5i shows the calibration curve between the distance and the width of the light spot on the camera. The angular position of the point light source can be determined from the centre position of the light spot on the camera (see the ‘Methods’). Figure 5b shows the 3D positioning of the point light source at different positions. For the calibration process, the yellow and green solid data points show the actual position and measured position of the light point source, respectively. The yellow and green circles show the actual position and measured position of a point source which had an unknown position a priori. The light distribution and the reconstructed images from the nominally-unknown point source are shown in Fig. 5j, k and Supplementary Fig. 26. The measured positions are consistent with the actual positions of the point source with a root-mean-square deviation of <0.16. The precision of the position tracking can be further improved by increasing the number of ommatidia of the BAC-eye and the bit depth of the CMOS camera. This 3D position tracking feature of the BAC-eye allows it to quantitatively locate a moving light source, which could be potentially implemented for advanced 3D phototaxic navigation and search applications, e.g., as a sensor to guide a robotic capsule endoscope to locate fluorophore-labelled lesions.

## Discussion

We have demonstrated a hybrid fabrication method that combines 3D printing and microfluidic-assisted moulding in order to generate a 3D BAC-eye that closely mimics the panoramic imaging capabilities of a natural compound eye. In contrast to conventional 2D fabrication techniques, microfluidic-assisted 3D printing produced precise microstructures inside of complex 3D micro-geometries, producing a BAC-eye that possesses many biomimetic components analogous to those contained in a natural compound eye, including corneal facet lenses, crystalline cones, rhabdoms and pigment cells. The BAC-eye was designed to both acquire full-colour 3D panoramic views and to subsequently seamlessly map the omnidirectional images to a planar imaging sensor, avoiding complex 3D photodetection techniques and bulky signal readout strategies. The imaging properties of the BAC-eye were characterized in detail to investigate the device’s capability to acquire panoramic views of surroundings and to track a point light source in 3D space. Wide-angle and full-colour panoramic images without aberrations were successfully reconstructed from the 2D images detected by the camera. Furthermore, precise 3D position tracking of a point light source was demonstrated without the need for complicated algorithms.

Because of the BAC-eye’s ability to seamlessly match to any 2D planar imaging sensor without the requirement of additional matching optics, we can improve the imaging performance (e.g., resolution and sensitivity) by increasing the number of the ommatidia and the filling factor of the compound eye. Additional details about the optimization of the BAC-eye can be found in Supplementary Note 7. The image from each ommatidium effectively contributes one pixel to the entire panoramic image, thus increasing the number of ommatidia present in the BAC-eye will proportionally increase the number of pixels in the resulting 2D image. In contrast, increasing the number of ommatidia in a compound eye that is manufactured by a less adaptable method such as deformable optoelectronics or nanowires requires a complete redesign of the entire imaging system, including complex matching optics and photodetectors. In principle, a full image can be formed independently from each ommatidium; therefore, 522 independent images with different view angles can be obtained simultaneously on one single planar image sensor. In the future, using imaging over fibre technology^53,54^, the speckle pattern obtained from each ommatidium could be reconstructed into an independent image, and an ultra-high-resolution panoramic image could be created. In addition, the BAC-eye normally functions in a receiving mode for panoramic imaging, i.e., collecting light from the top surface and transmitting the light to the bottom to form an image on a planar image sensor. However, the BAC-eye could also function in an emitting mode by replacing the image sensor with a 2D display, e.g., with a liquid crystal display, for potential applications in planetarium projection systems^55^ and volumetric 3D displays^56^. On a fundamental level, the BAC-eye may also be useful as a biomimetic model for natural compound eyes, allowing scientists to study and test the mechanisms behind insect vision and perception. In addition, due to the miniaturized design and scalability of the BAC-eye, it could be adopted by fields such as micro-robotics where it can be utilized for applications including 3D endoscopic vision in industrial and medical inspections; the BAC-eye may also be useful for machine vision for functional human–robot interactions, and improving 3D displays.

## Methods

### Fabrication process for the BAC-eye

Supplementary Fig. 1 shows the fabrication process for the BAC-eye, as follows: (1) A mould consisting of an open hemispherical pit and 522 cylindrical microholes arranged omnidirectionally on the bottom of the pit was designed by computer-aided design (CAD) software and 3D printed by a 3D printer with a printing resolution of 10 μm (nanoArch® P140, BMF Precision Technology Co., China). (2) Acrylate resin (Aroh Alona, China) was added to the pit of the mould. The mould was placed in vacuum at –0.1 MPa for 10 min to remove any microbubbles from the acrylate resin. (3) The mould was then spun at 1500 rpm for 4 min. (4) After the spinning stopped, the mould was placed in a dark environment for 30 min. (5) The mould was then exposed to UV light for 15 min to cure the acrylate resin. (6) A hemispherical substrate consisting of 522 hollow pipelines was designed and 3D printed using a photosensitive polymer dyed with Sudan Black 3 (Sigma-Aldrich, USA) at a concentration of 1500 μg/mL with the same 3D printer (nanoArch® P140, BMF Precision Technology Co., China). The design of the pipeline structure, which acts as optical waveguides, is discussed in Supplementary Fig. 3. (7) The hemispherical substrate was inserted into the pit of the mould. The six auxiliary supports around the hemisphere of the substrate (Fig. 1g) and the six slots on the surface of the mould (Supplementary Fig. 2) were used to align the pipelines in the substrate with the microholes in the mould. (8) The entire mould plus hemispherical substrate structure was immersed into liquid-state RTV silicone (Part A: phenyl(chlorophenyl)siloxane-dimethylsiloxane copolymer, vinyldimethylsiloxane terminated and Part B: methylhydrosiloxane-phenylmethylsiloxane copolymer, hydride terminated; the weight ratio of Part A and Part B is 1:1) (Gelest, Inc.) and evacuated at –0.1 MPa for 20 min to ensure that the RTV silicone completely filled in the pipelines and the microholes. (9) The RTV silicone was then cured at 55 °C for 4 h. (10) The fully formed BAC-eye was separated from the mould.

### Characterization of the materials

In order to reduce the optical crosstalk due to light leakage in the waveguides of the ommatidia, the photosensitive polymer used for the supporting structures of the BAC-eye was mixed with Sudan Black 3 solvent dye. Four 100-μm slices were 3D printed using the prepared photosensitive polymer. The optical density of the slices was measured using a spectrophotometer (LAMBDA 1050, PerkinElmer, Inc., USA). When the dye concentration was 1500 μg/mL, the optical density, in the wavelength range from 400 to 800 nm, was above 3.3. The optical transmission spectrum of the 10-mm-thick RTV silicone was also measured using the spectrophotometer. The contact angle, *θ*_*e*_, of an acrylate resin droplet on the flat photosensitive polymer substrate and the curvature of the microlenses were measured by an optical contact angle meter (SL200B, KINO Scientific Instrument Inc., USA).

### Panoramic imaging system

The experimental setup of the panoramic imaging system is illustrated in Fig. 4b. A broadband tungsten-halogen light source (SLS201L/M, Thorlabs, Inc., USA) was used to generate white light. 450 and 633 nm semiconductor lasers (MDL-E-450 and MRL-III-633L, CNI Laser, China) were used to generate blue and red light. The collimated light via fiber collimators (F810FC-543, Thorlabs, Inc., USA) illuminated masks with square, cross and triangle patterns, respectively. Each mask was placed in front of the compound eye with a distance of 8 mm. A telecentric lens (1X, 40 mm WD CompactTL™, Edmund Optics Inc., USA), whose aperture was *f*/11, was used to magnify the image at the flat base of the eye. The image was recorded using a CMOS camera (EO-18112, Edmund Optics Inc., USA).

### Reconstruction of a panoramic image

A panoramic image was directly mapped from the flat bottom of the BAC-eye onto a camera. Each sub-image projected from the flat end of the waveguide/pipeline onto the camera was homogenized by taking the average value of every sub-image. A block of *N* × *N* pixels in the sub-image was used to represent a sub-view of a particular ommatidium, that is oriented with the polar angle, *α*, and azimuthal angle, *β*. The proximal end of the waveguide was centred at the position of (*x*_Bottom_, *y*_Bottom_) in the plane ℝ^2^ (Supplementary Fig. 22). The relation between the orientation of the ommatidium and the centre of the proximal end of the waveguide can be written as

1 $$\alpha =\frac{2\sqrt{{x}_{{{{{{\rm{Bottom}}}}}}}^{2}+{y}_{{{{{{\rm{Bottom}}}}}}}^{2}}}{D}\times 90^\circ$$

2 $$\beta = \left\{\begin{array}{c}{{{{{\mathrm{arctan}}}}}}(\frac{{y}_{{{{{{\rm{Bottom}}}}}}}}{{x}_{{{{{{\rm{Bottom}}}}}}}}),x\ge 0\\ \arctan (\frac{{y}_{{{{{{\rm{Bottom}}}}}}}}{{x}_{{{{{{\rm{Bottom}}}}}}}})+180^\circ ,x < 0\end{array}\right.$$

The sub-view is then used as a part of the panoramic view. The corresponding centre position of the sub-view in the 3D Euclidean space ℝ^3^ is (*x*_3D_, *y*_3D_, *z*_3D_), which can be expressed as

3 $${x}_{3{{{{{\mathrm{D}}}}}}}=\frac{L{{{{{\mathrm{tan }}}}}}\left(\alpha \right){{{{{\mathrm{cos }}}}}}\left(\beta \right)}{\sqrt{1+{{{{{{\mathrm{tan }}}}}}}^{2}\left(\alpha \right)}}$$

4 $${y}_{3{{{{{\rm{D}}}}}}}=\frac{L{{{{{\mathrm{tan }}}}}}\left(\alpha \right){{{{{\mathrm{sin }}}}}}\left(\beta \right)}{\sqrt{1+{{{{{{\mathrm{tan }}}}}}}^{2}\left(\alpha \right)}}$$

5 $${z}_{3{{{{{\rm{D}}}}}}}=\frac{L}{\sqrt{1+{{{{{{\mathrm{tan }}}}}}}^{2}\left(\alpha \right)}}$$

where *L* is the nominal viewing distance assuming an observer is located at the origin of the BAC-eye. Since the panoramic image is a virtual image of the real object, the nominal viewing distance *L* is defined as a dimensionless parameter as:

6 $$L=\frac{N}{2{{{{{\mathrm{tan }}}}}}\left(\frac{\triangle \alpha }{2}\right)}$$

where *N* is the pixel number of the sub-view, Δ*α* is the difference in polar angle of the orientation between the two adjacent ommatidia (with the orientation at the same azimuthal angle).

### Positioning of a point light source

When a point light source illuminates a BAC-eye, the proximal ends of the corresponding ommatidia also become illuminated. The intensity of the light output from each ommatidium was homogenized and the light spot on the camera was fitted with a Gaussian function as follows

7 $$G(x,y)={I}_{{{{{{\rm{max }}}}}}}\left({{{{{{\rm{e}}}}}}}^{-\frac{{\left(x-{x}_{{{{{{\rm{max }}}}}}}\right)}^{2}}{2{\sigma }^{2}}}+{{{{{{\rm{e}}}}}}}^{-\frac{{\left(y-{y}_{{{{{{\rm{max }}}}}}}\right)}^{2}}{2{\sigma }^{2}}}\right)$$

where *I*_max_ is the peak intensity of the distribution, *x*_max_ and *y*_max_ are the position of the peak and *σ* is the width of the Gaussian distribution. The distance between the point light source and the origin of the BAC-eye (*L*_P_) is related to the width of the Gaussian distribution and can be calibrated with a point light source with a known distance *L*_P_. In this experiment, a logarithmic calibration function was obtained as:

8 $$\sigma =\frac{{p}_{1}}{1+{{\mathrm e}^{-{p}_{2}{({L}_{p}-{{p}_{3})}}}}}+{p}_{4}$$

where *p*_1_, *p*_2_, *p*_3_ and *p*_4_ are the fitting parameters. The measured distance *L*_P_ can be readily obtained by measuring the width of the Gaussian distribution of the light spot on the camera as:

9 $${L}_{{{{{{\rm{P}}}}}}}={p}_{3}-\frac{1}{{p}_{2}}{{{{{\rm{log }}}}}}\left(\frac{{p}_{1}}{\sigma -{p}_{4}}-1\right)$$

The peak position of the Gaussian distribution reveals the orientation of the point light source, which can be calculated by using Eqs. (1) and (2). Finally, the position of the spotlight source can be determined using Eqs. (3)–(5).

## Supplementary information

Supplementary Information

## Data Availability

The data that support the findings of the study are available from the corresponding author upon reasonable request.

## Code availability

The codes used for panoramic imaging are available from the main developer (daibo@usst.edu.cn) upon reasonable request.
