# Supplementary material for: Biomimetic apposition compound eye fabricated using microfluidic-assisted 3D printing
Source: Nat Commun. 2021 Nov 9;12:6458. doi: 10.1038/s41467-021-26606-z (PMC8578215; doi:10.1038/s41467-021-26606-z)
Supplement: Supplementary file 1 — Supplementary Information [file 41467_2021_26606_MOESM1_ESM.pdf]

Supplementary Information

Biomimetic apposition compound eye fabricated using microfluidic-assisted 3D printing

Bo Dai[1,†], Liang Zhang[1,†], Chenglong Zhao[2,3†], Hunter Bachman[4], Ryan Becker[5], John Mai[6], Ziao Jiao[1], Wei Li[1], Lulu Zheng[1], Xinjun Wan[1], Tony Jun Huang[4*], Songlin Zhuang[1], and Dawei Zhang[1*]

[1] Engineering Research Center of Optical Instrument and System, the Ministry of Education, Shanghai Key Laboratory of Modern Optical System, University of Shanghai for Science and Technology, Shanghai, 200093, China

[2] Department of Physics, University of Dayton, Dayton, OH 45469, USA

[3] Department of Electro-Optics and Photonics, University of Dayton, Dayton, OH 45469, USA

[4] Department of Mechanical Engineering and Materials Science, Duke University, Durham, NC 27709, USA

[5] Department of Biomedical Engineering, Duke University, Durham, NC 27709, USA

[6] Alfred E. Mann Institute for Biomedical Engineering, University of Southern California, Los Angeles, CA 90089, USA

† These authors contributed equally: Bo Dai, Liang Zhang, Chenglong Zhao

* Correspondence and requests for materials should be addressed to T.J.H. (email: tony.huang@duke.edu) or D.Z. (email: dwzhang@usst.edu.cn)

Supplementary Figures

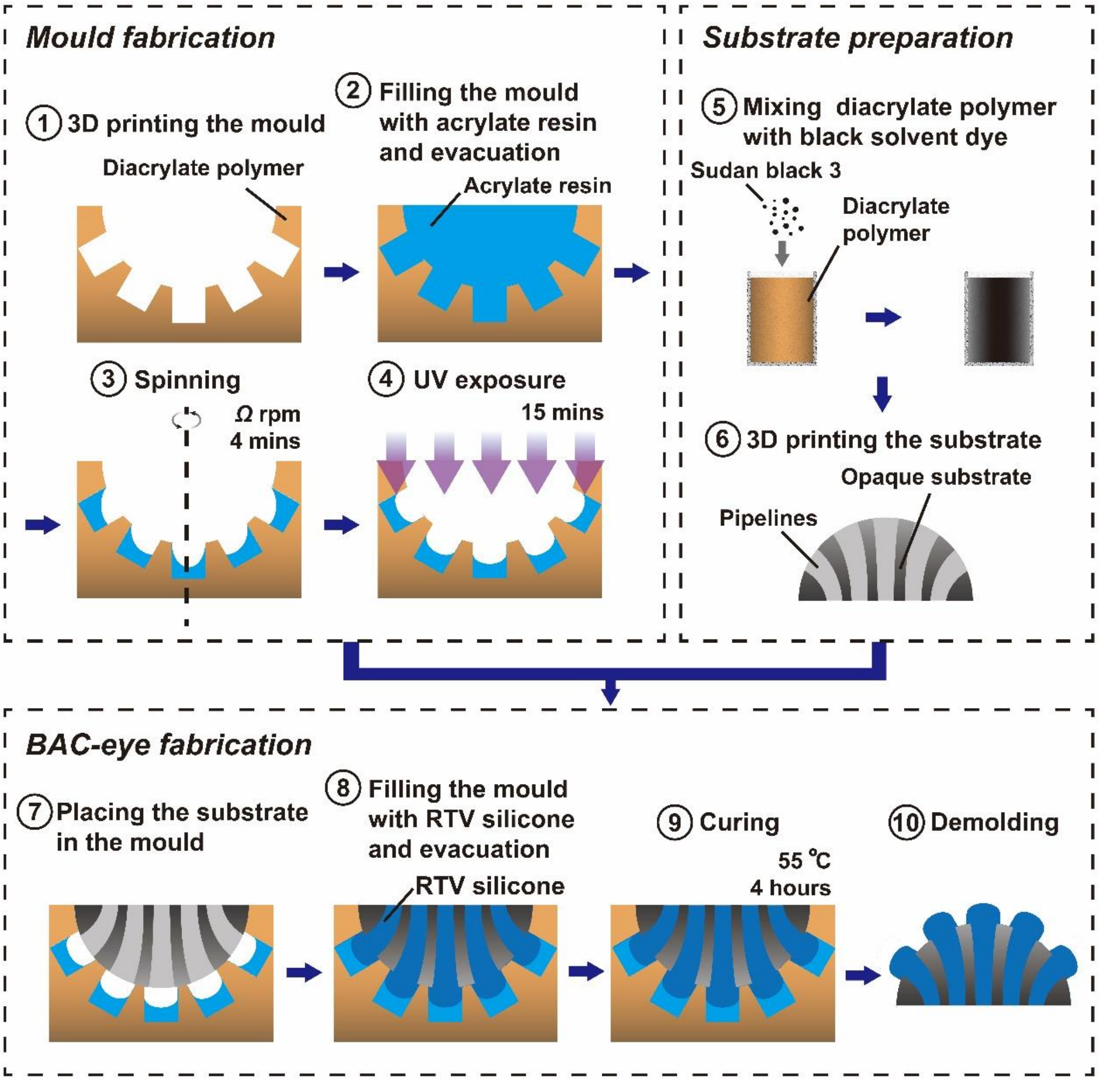

Supplementary Fig. 1. Fabrication procedure of the mould and the BAC-eye. 1-4) Microlens mold formation. The 3D printed mold is filled with acrylate resin, spun to form the desired concavity of the lenses and allowed to cure. 5-6) 3D printed compound eye using a diacrylate polymer with a high optical density dye. 7-10) Formation of the ommatidia for the BAC-eye; after placing the compound eye in the mold, silicon is poured into the channel and allowed to cure, forming the waveguides and microlenses for the ommatidia.

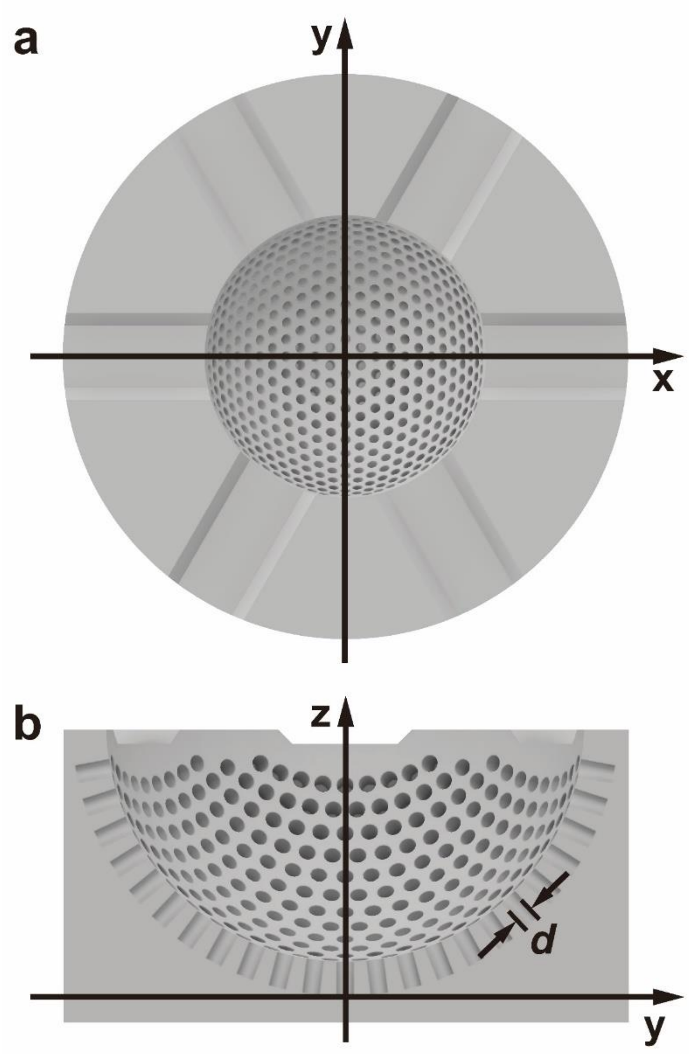

Supplementary Fig. 2. 3D model of the mould. a, Top view of the substrate. **b,** Cross section of the substrate.

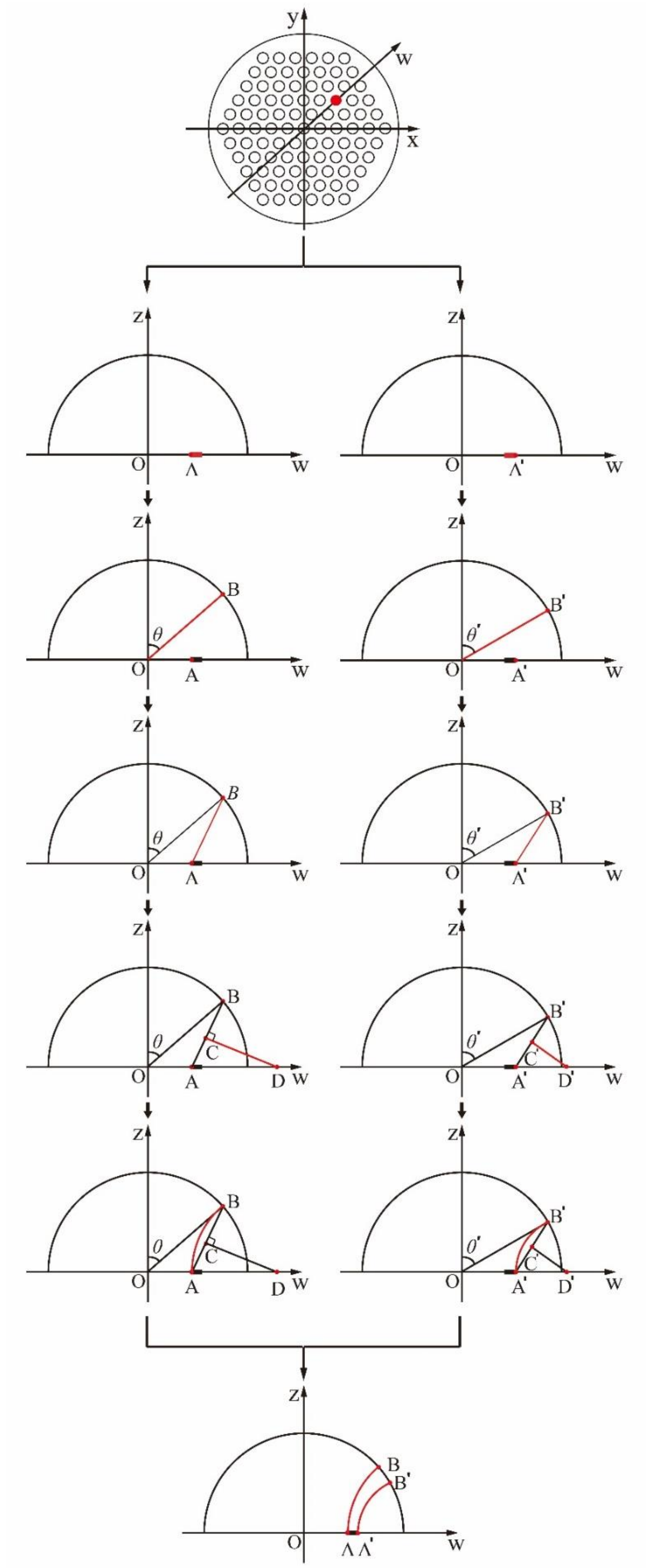

Supplementary Fig. 3. Design procedure for the substrate. The distribution pattern of the waveguide ends are first determined on the flat base of the hemisphere. Then, hollow channels are designed within the hemisphere.

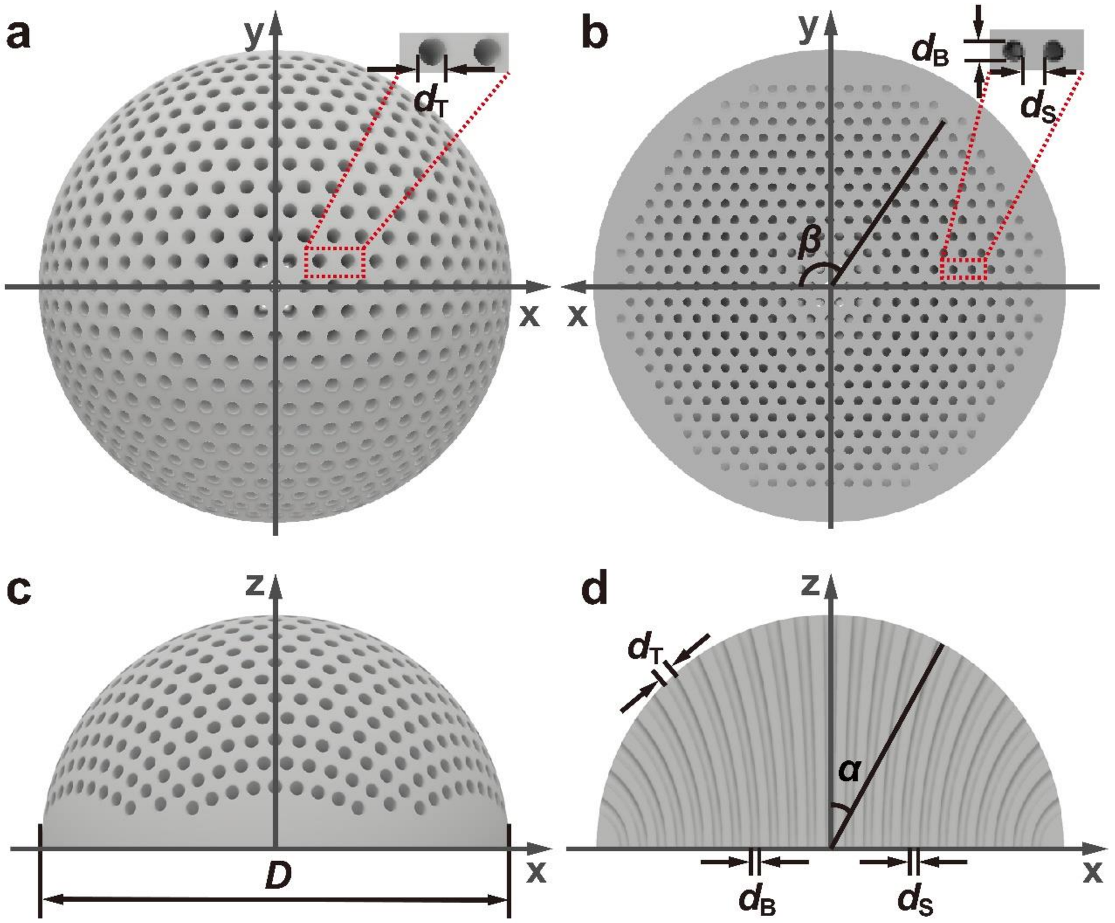

Supplementary Fig. 4. 3D model of the substrate. a, Top view of the substrate. **b,** Bottom view of the substrate. **c,** Side view of the substrate. **d,** Cross section of the substrate.

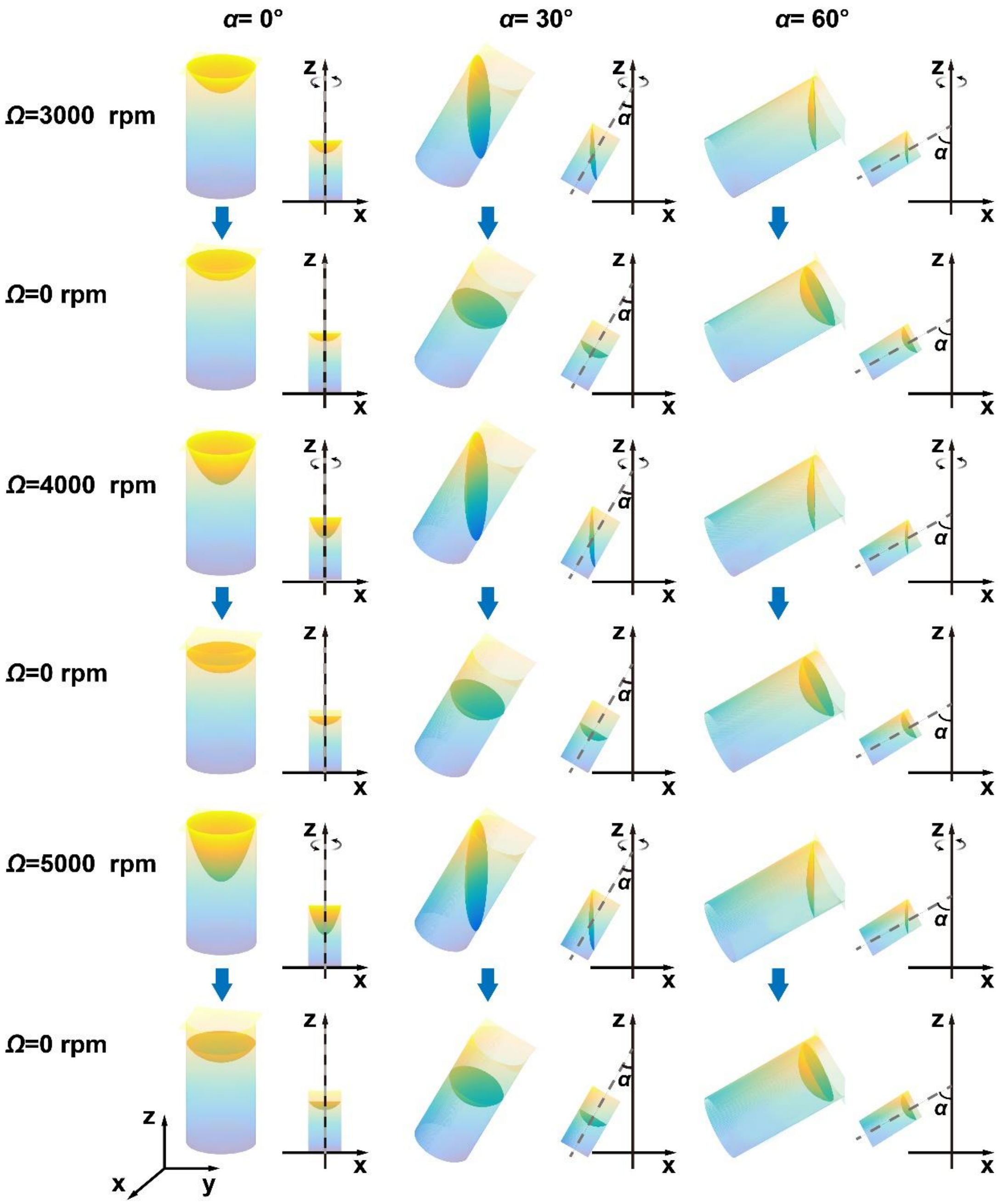

Supplementary Fig. 5. Simulation of the surface profile of the acrylate resin in microholes with different orientations during the spinning process at different spin rates (in dynamic equilibrium) and after spinning (in static equilibrium). During spinning, a portion of the acrylate resin ascends up the side of the microhole and is ejected. After spinning, the surface of the acrylate resin becomes concave due to the surface tension. The amount of acrylate resin remaining in the microhole decreases as the spin speed increases.

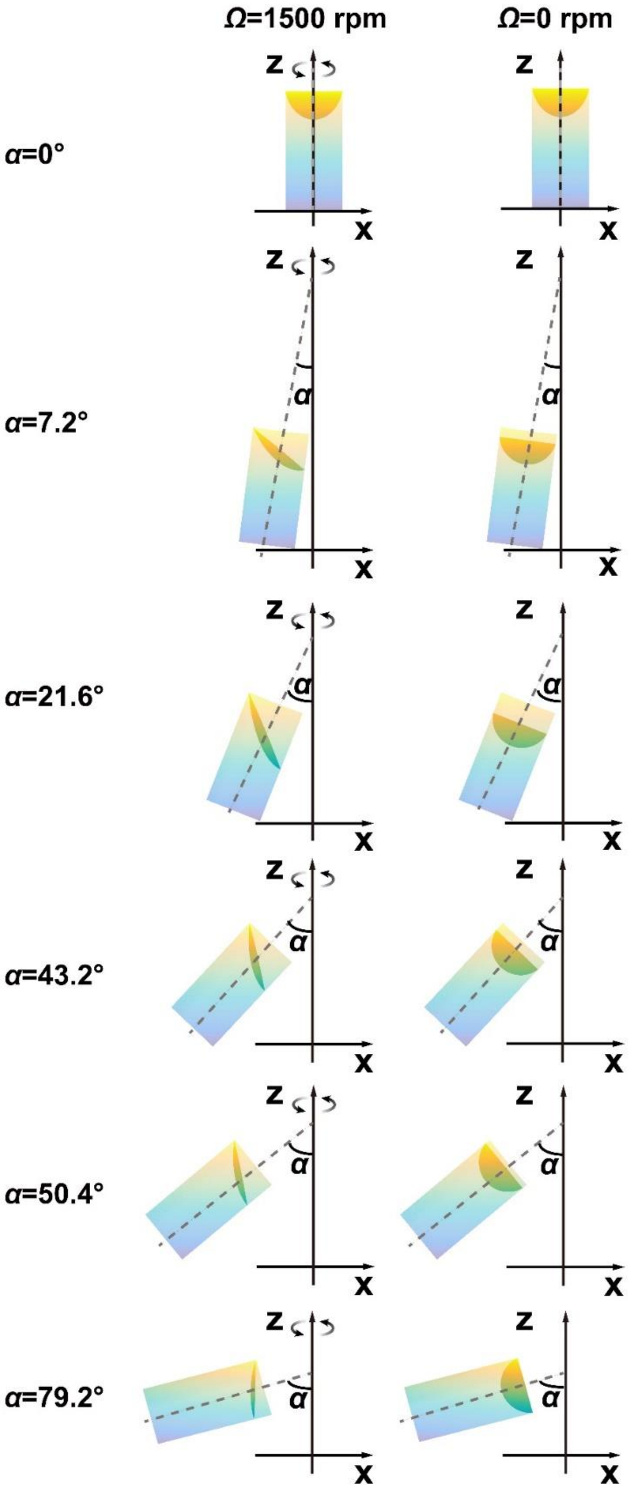

Supplementary Fig. 6. Simulation of the surface profile of the acrylate resin in microholes with different orientations simulated both during spinning with a spin rate of 1500 rpm (left column) and after spinning (right column). In the off-axis microholes (*α≠0º*) closer to the centre, more acrylate resin is ejected during spinning, while less acrylate resin remains in the microholes after spinning.

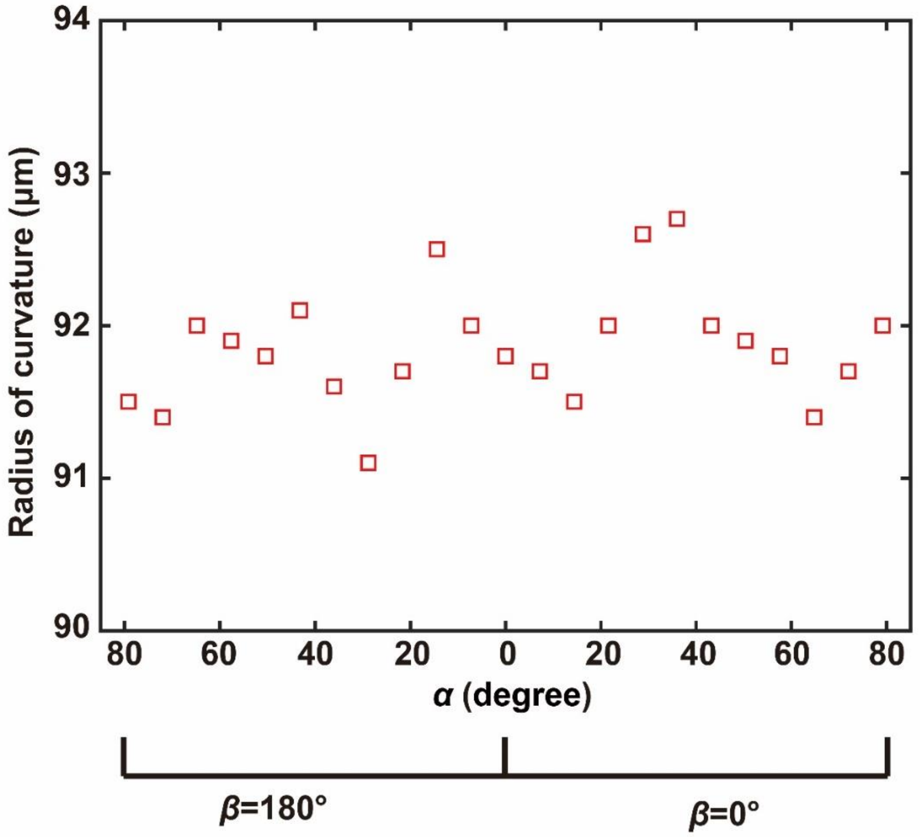

Supplementary Fig. 7. Measurement of the radius of curvature of the microlenses on the ommatidia at different orientations. The radius of curvature is 91.9±0.8 μm, demonstrating a uniform profile for the microlenses.

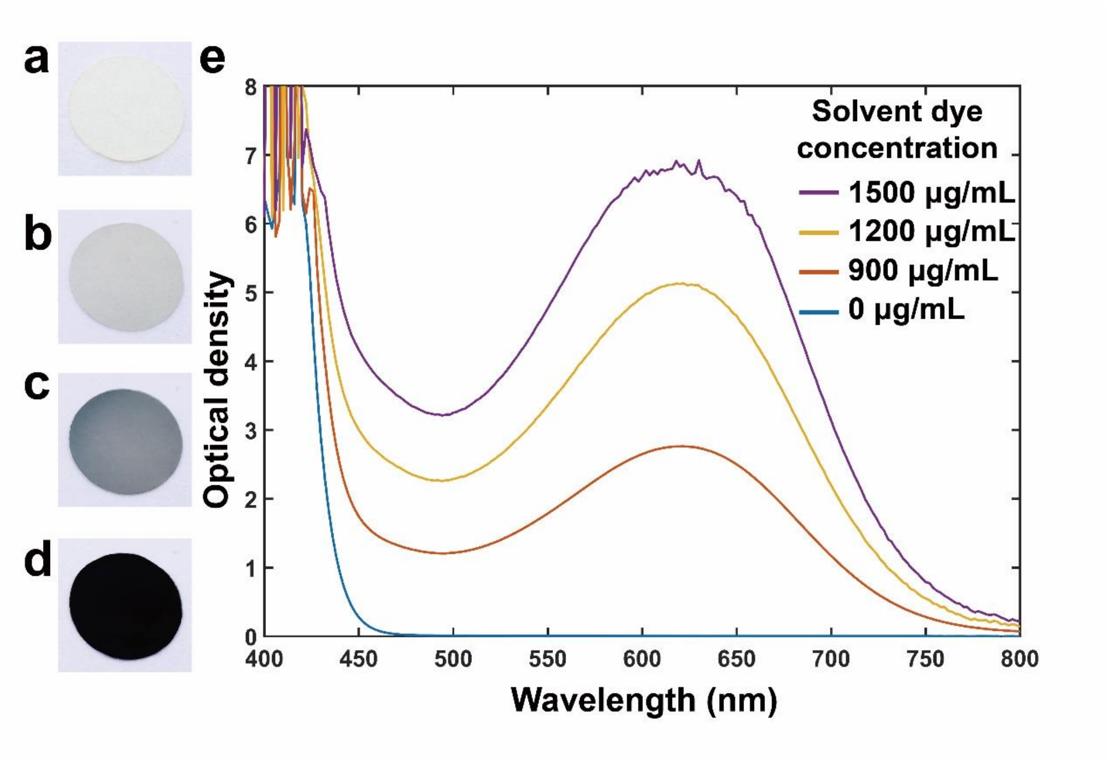

Supplementary Fig. 8. Evaluation of the optical density of the photosensitive polymer dyed with Sudan Black 3 solvent dye. a, A cured, 100 µm thick polymer slice without any solvent dye. **b-d,** Cured, 100 µm thick polymer slices mixed with 900 µg/mL, 1200 µg/mL, and 1500 µg/mL solvent dye, respectively. **e,** Optical density of the photosensitive polymer and the Sudan Black 3 solvent dye mixture at different concentrations over the visible spectrum.

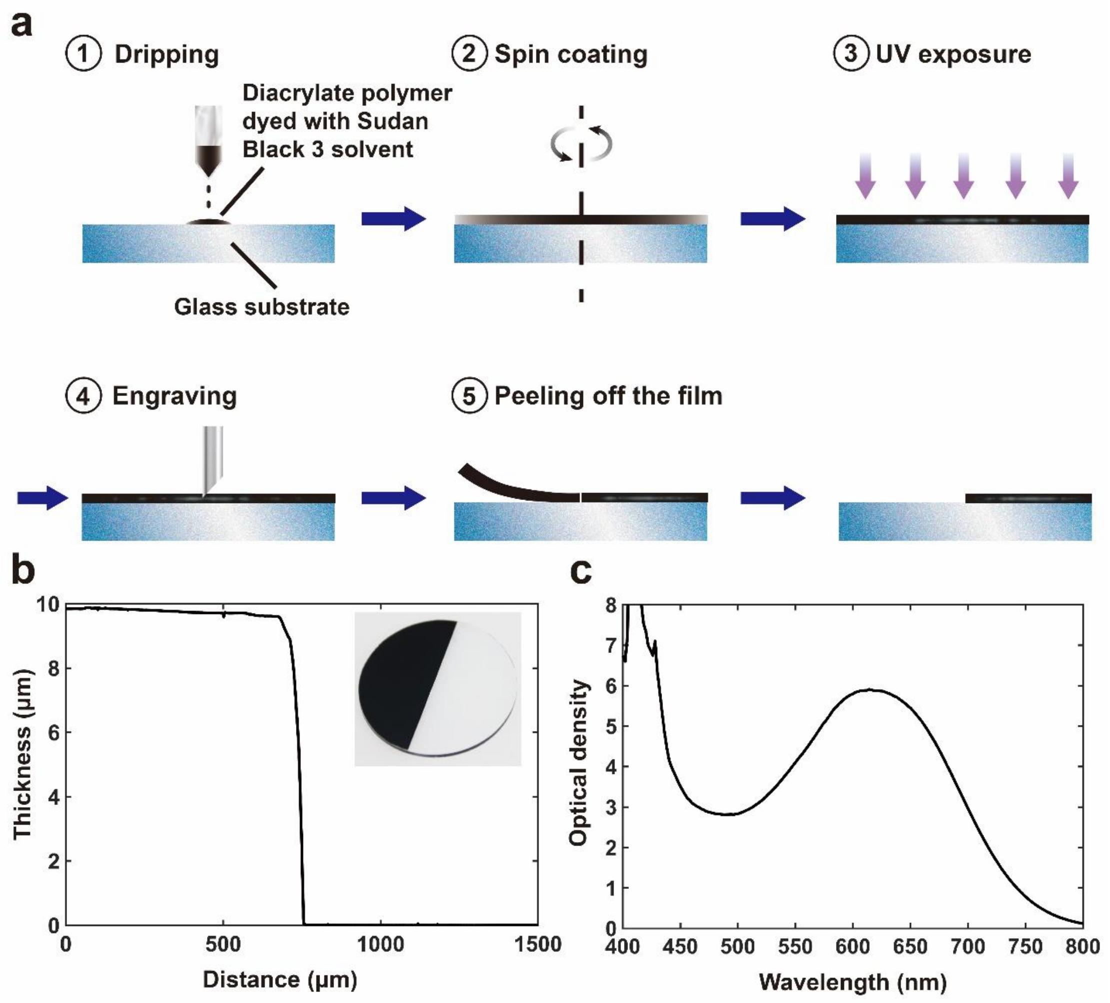

Supplementary Fig. 9. Evaluation of the optical density of the thin photosensitive polymer film. a, Preparation of the thin photosensitive polymer film. For the UV exposure, 405 nm LED was used and the UV exposure energy was 465 mJ/cm^2. **b,** Measurement of the film thickness. Inset: A photo of the prepared photosensitive polymer film. **c,** The optical density of the film.

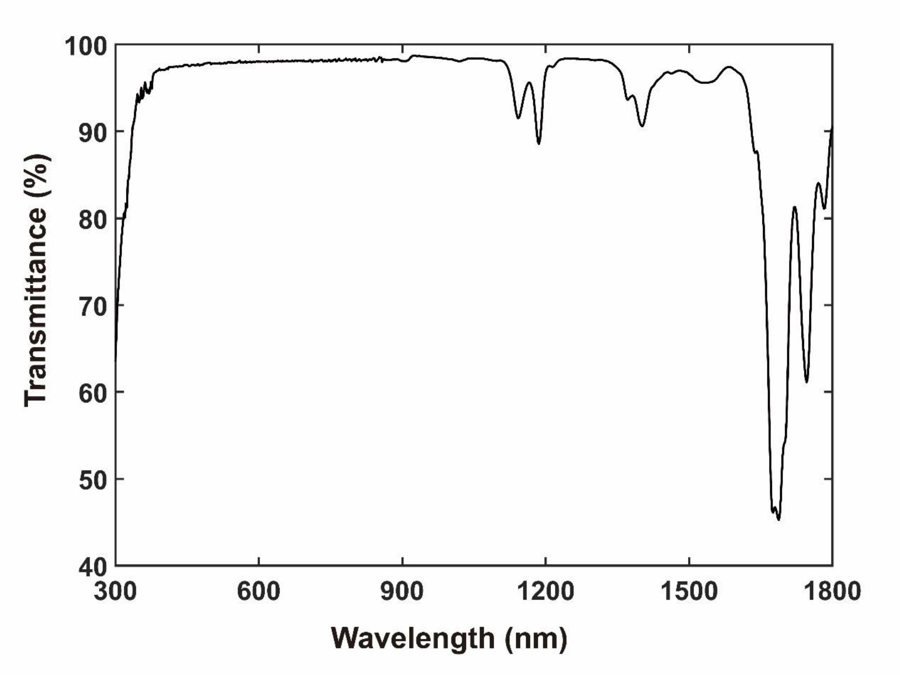

Supplementary Fig. 10. The optical transmission spectrum of RTV silicone with a thickness of 10 mm. The RTV silicone has high transmittance (>97%) over the entire visible spectrum (400 nm to 800 nm) and good transmission characteristics (transmittance > 87.5%) in the infrared region (800 nm to 1600 nm).

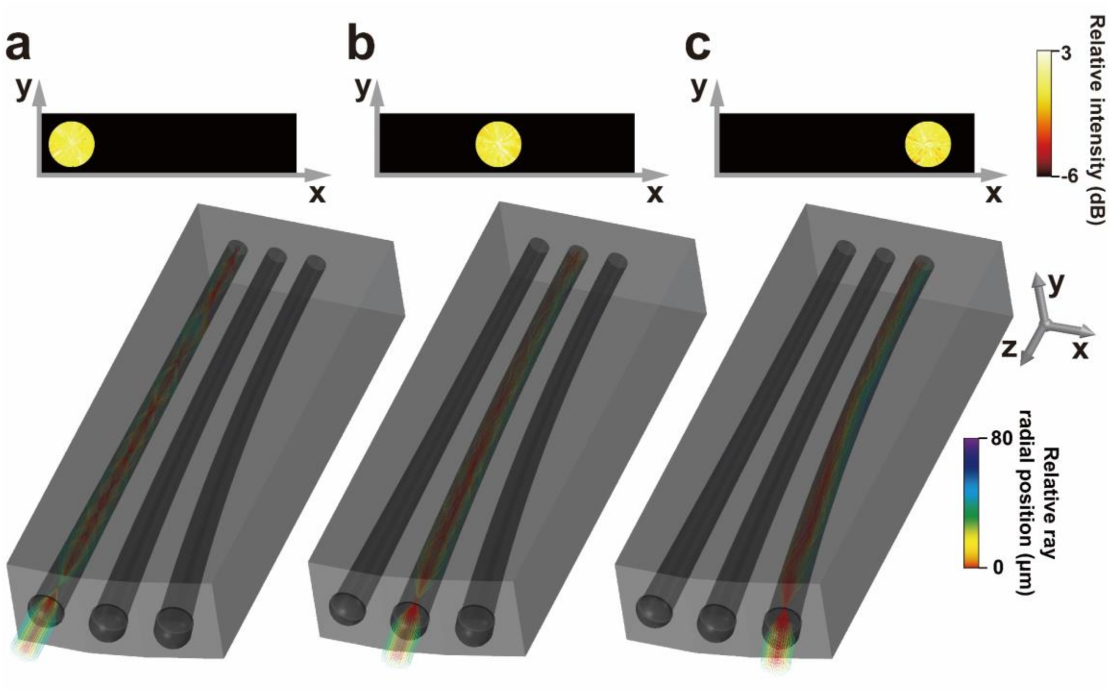

Supplementary Fig. 11. Simulation of the optical crosstalk among three central ommatidia. The light is incident on the microlens of the ommatidium with orientations of **a,** (*α=0º, β=0º*), **b,** (*α=7.2º, β=0º*) and **c,** (*α=14.4º, β=0º*). The intensity distribution at the proximal end of the waveguide is relative to the ommatidium that the light is incident on.

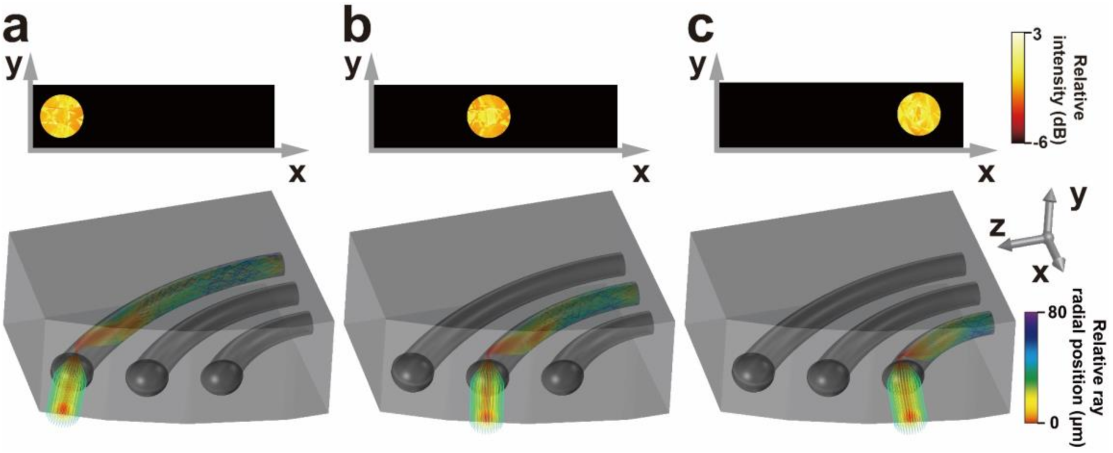

Supplementary Fig. 12. Simulation of the optical crosstalk among three highly-curved ommatidia. The light is incident on the microlens of the ommatidium with the orientation of **a,** (*α=64.8º, β=0º*), **b,** (*α=72º, β=0º*) and **c,** (*α=79.2º, β=0º*). The intensity distribution at the proximal end of the waveguide is relative to the ommatidium that the light is incident on.

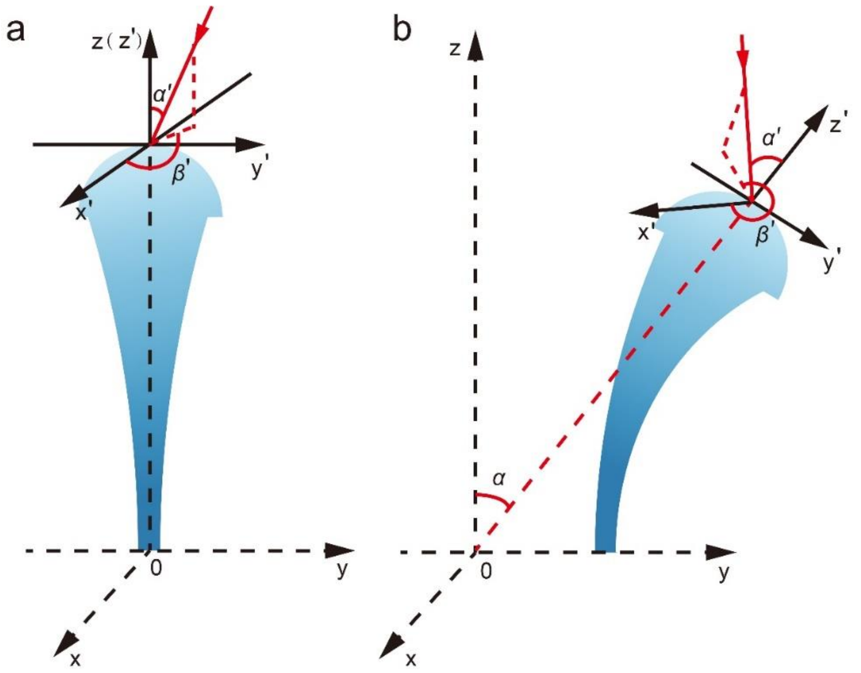

Supplementary Fig. 13. The coordinate system used for angular sensitivity measurements. The *xyz*-coordinate system is used to determine the position and the orientation of the ommatidia. The polar angle, *α*, and azimuthal angle, *β*, describe the orientation of each ommatidium. The polar angle, *α'*, and azimuthal angle, *β'*, in the *x'y'z'*-coordinate system are used to describe the incident angle of the incident light. The angle of intersection between the *z*-axis and *z'*-axis is the polar angle, *α*, that determines the orientation of each ommatidium.

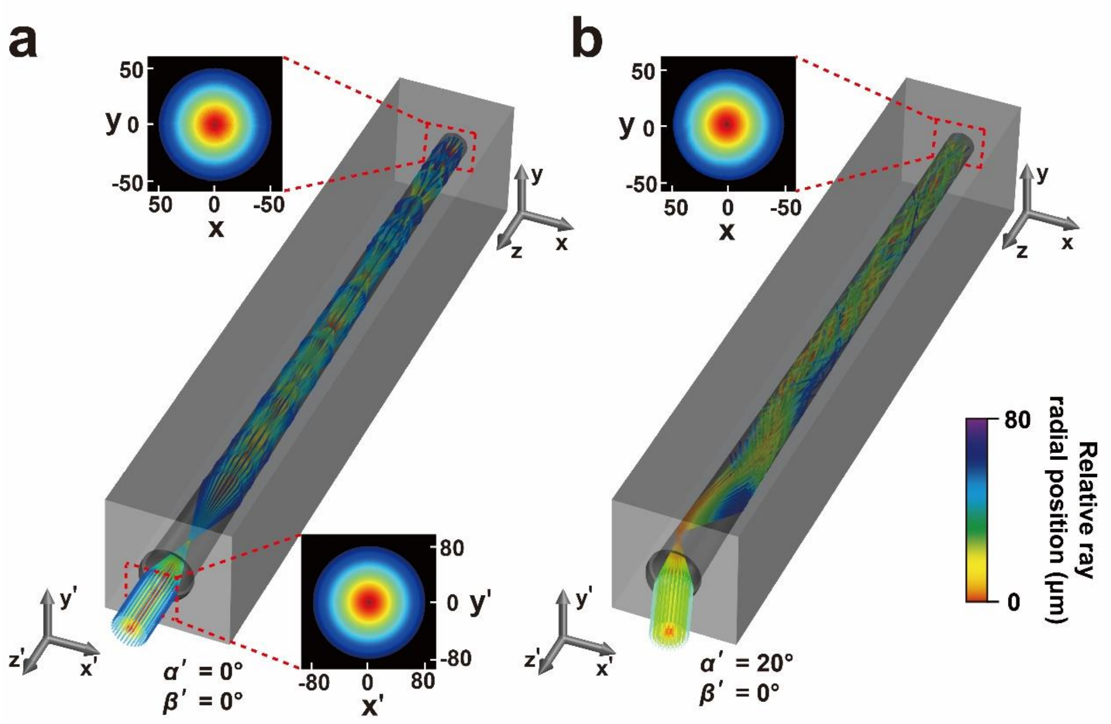

Supplementary Fig. 14. Simulation of optical ray tracing in the centre ommatidium (*α*=*0º*). a, Normal incidence, *i.e.* incident angle of *α'=0º* and *β'=0º*. **b,** Oblique incidence with an incident angle of *α'=20º* and *β'=0º*.

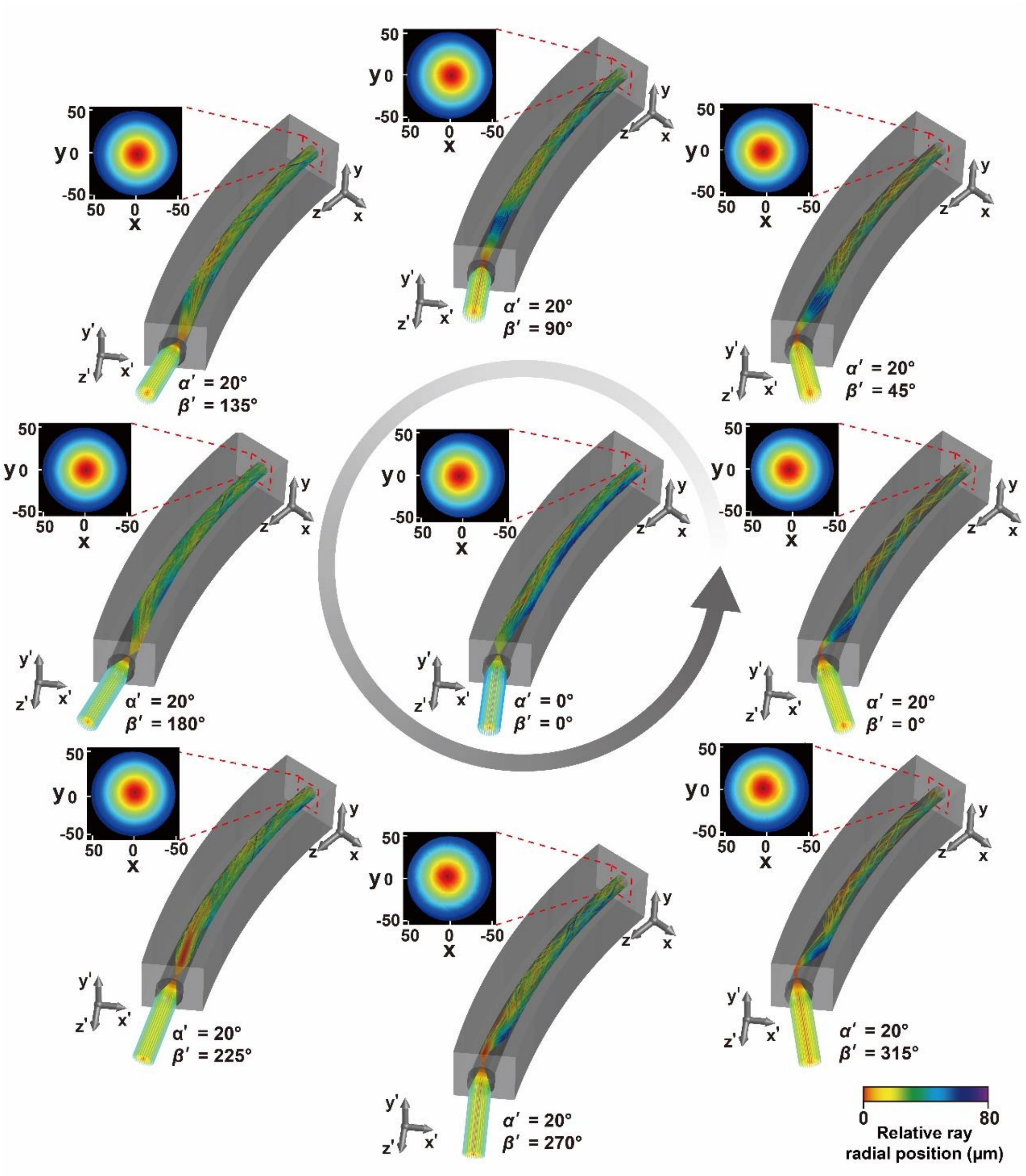

Supplementary Fig. 15. Simulation of optical ray tracing in the ommatidium with an orientation of (*α=36º, β=0º*) when the collimated beam is incident on the microlens at different incident angles.

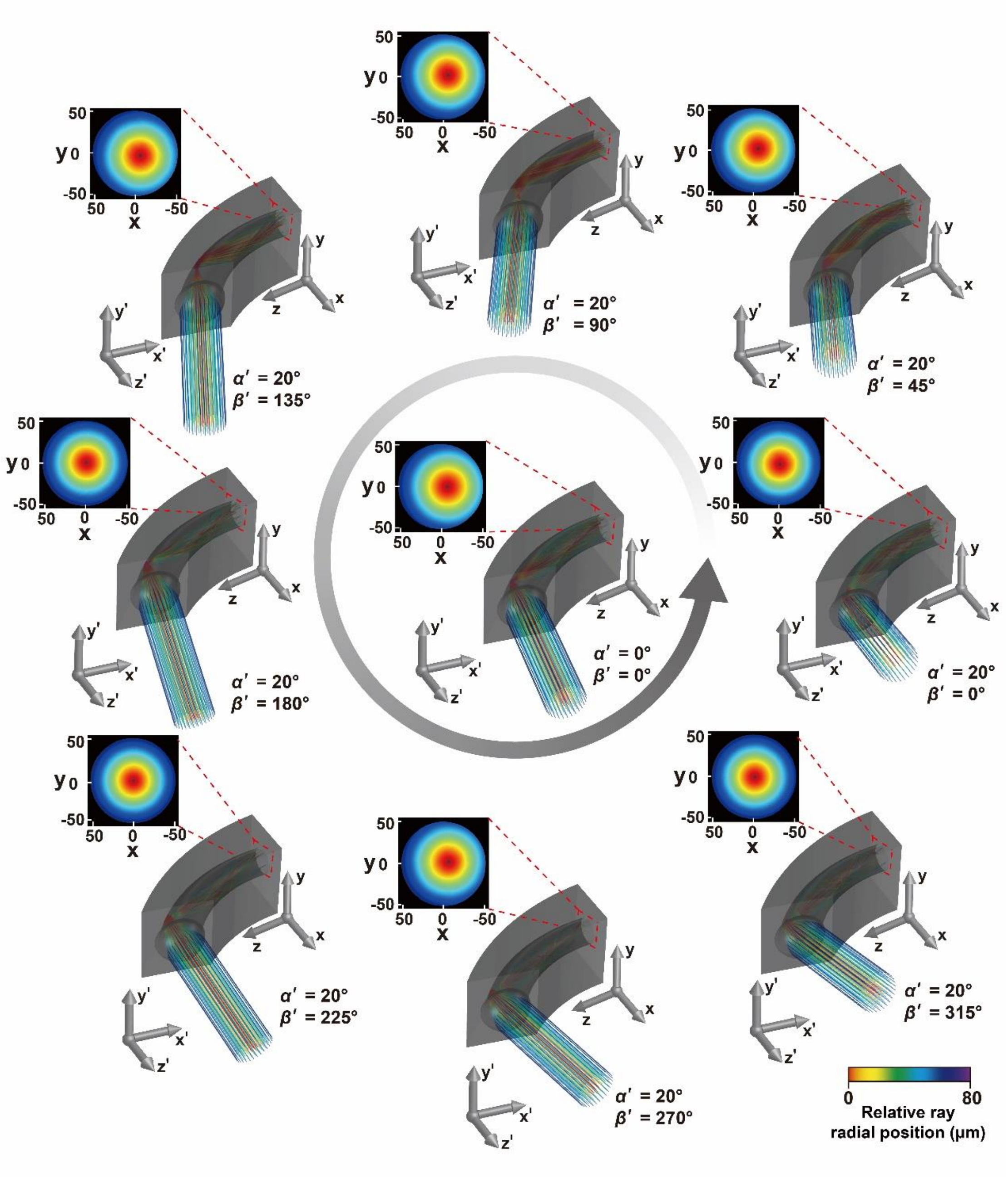

Supplementary Fig. 16. Simulation of optical ray tracing in the ommatidium with an orientation of (*α=79.2º, β=0º*) when the collimated beam is incident on the microlens with different incident angles.

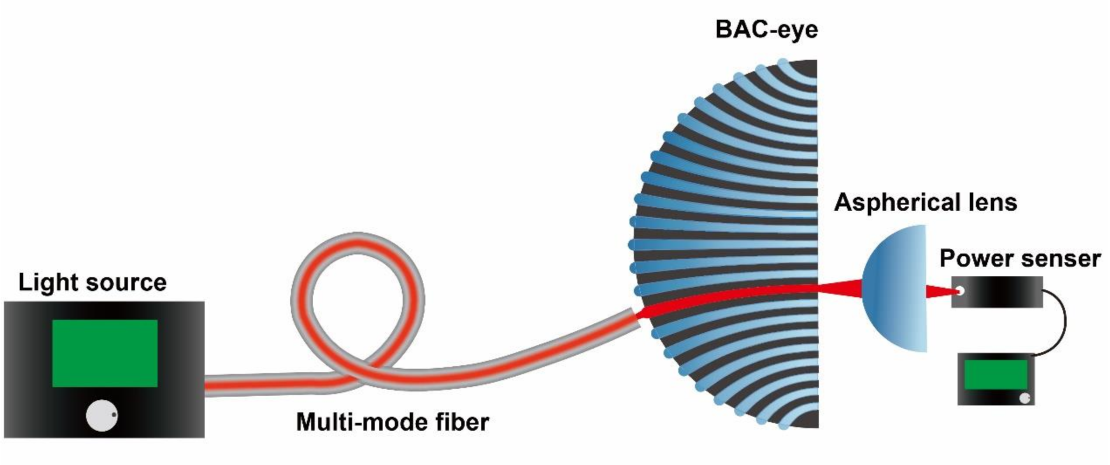

Supplementary Fig. 17. Experimental setup for the measurement of the optical loss in the ommatidia. Light is generated by a tungsten-halogen light source and outputs from a multi-mode fibre. The tip of the fibre is close to (2 mm) the microlens of the ommatidium. The light is normally incident on the microlens. Behind the eye, the output light from the end of the waveguide is collected by an aspherical lens and measured by a photodiode power source. The optical loss is calculated by comparing the optical power measured directly from the tip of the multi-mode fibre and from the end of the waveguide via the ommatidium.

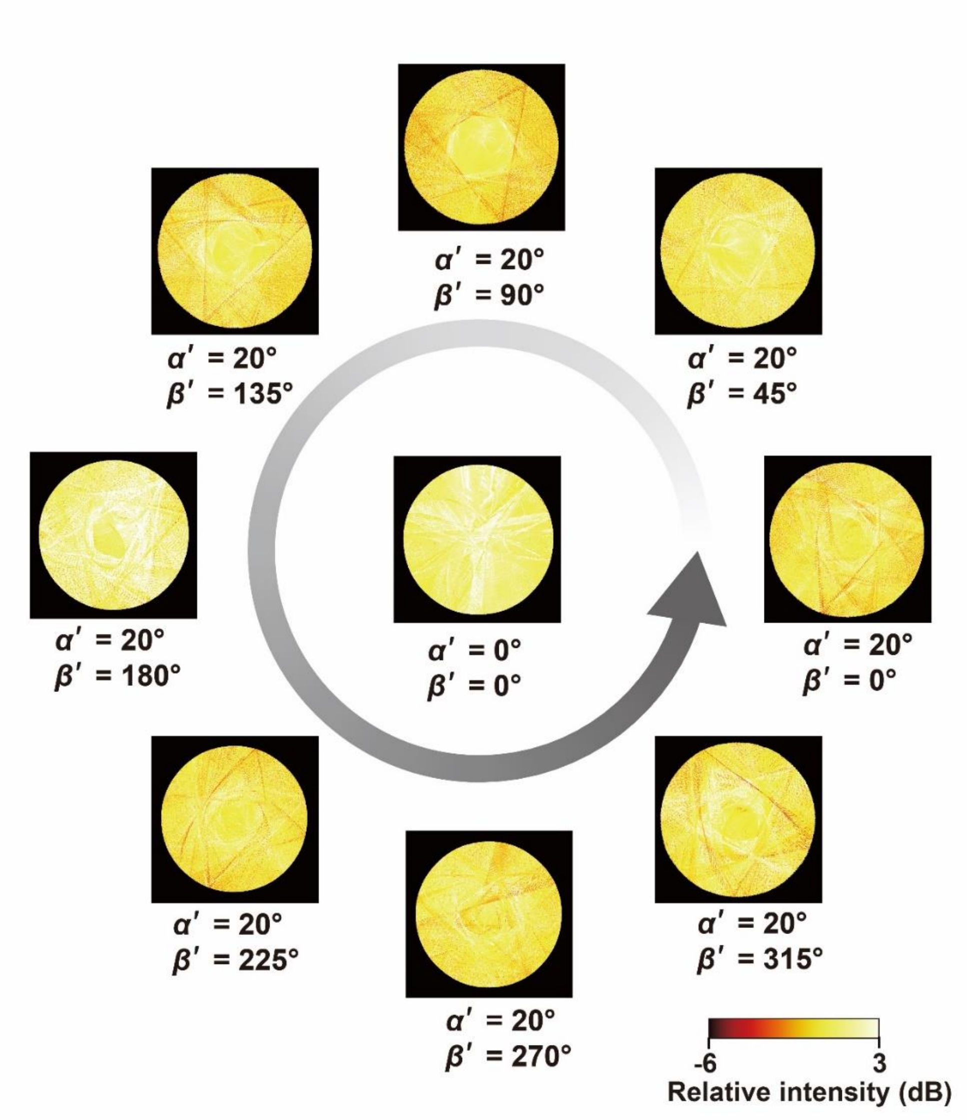

Supplementary Fig. 18. Light intensity distribution at the proximal end of the waveguide in the ommatidium with an orientation of (*α=36º, β=0º*) when the light is incident on the ommatidium at different incident angles. The intensity distribution at the proximal end of the waveguide is relative to the incidence angle of the light.

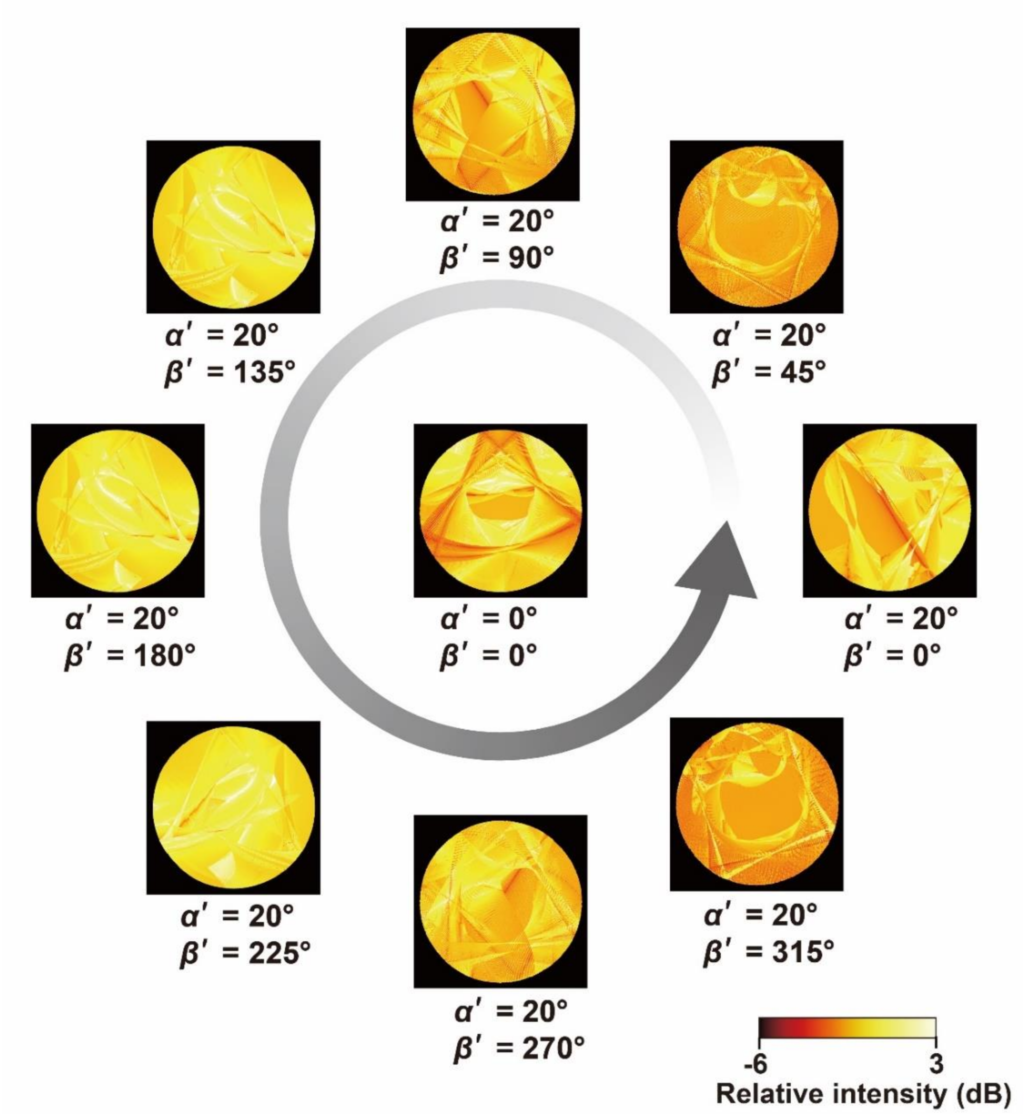

Supplementary Fig. 19. Light intensity distribution at the proximal end of the waveguide in the ommatidium with an orientation of (*α=79.2º, β=0º*) when the light is incident on the ommatidium at different incident angles. The intensity distribution at the proximal end of the waveguide is relative to the incidence angle of the light.

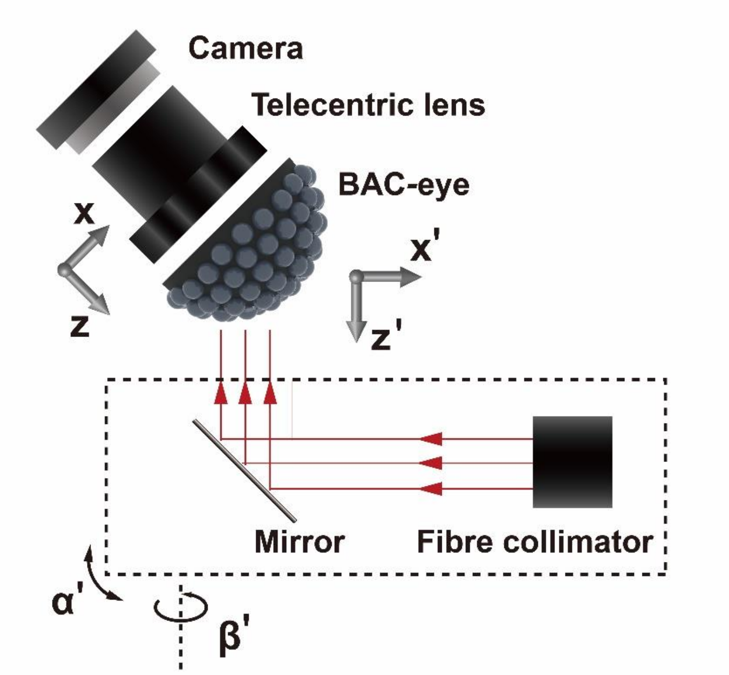

Supplementary Fig. 20. The experimental setup for measuring the angular sensitivity function. A collimated beam is generated from a light source via a fibre collimator. The collimated beam is incident on the microlens of an ommatidium with different incident angle (polar angle, *α'*, and azimuthal angle, *β'*). The light is collected via a telecentric lens by a CMOS camera. An average value representing the output intensity is calculated based on the 80×80 pixels in the sub-image in the sub-image from the corresponding ommatidium. The angular sensitivity function is then obtained by summarizing the output intensity with the different incident angles.

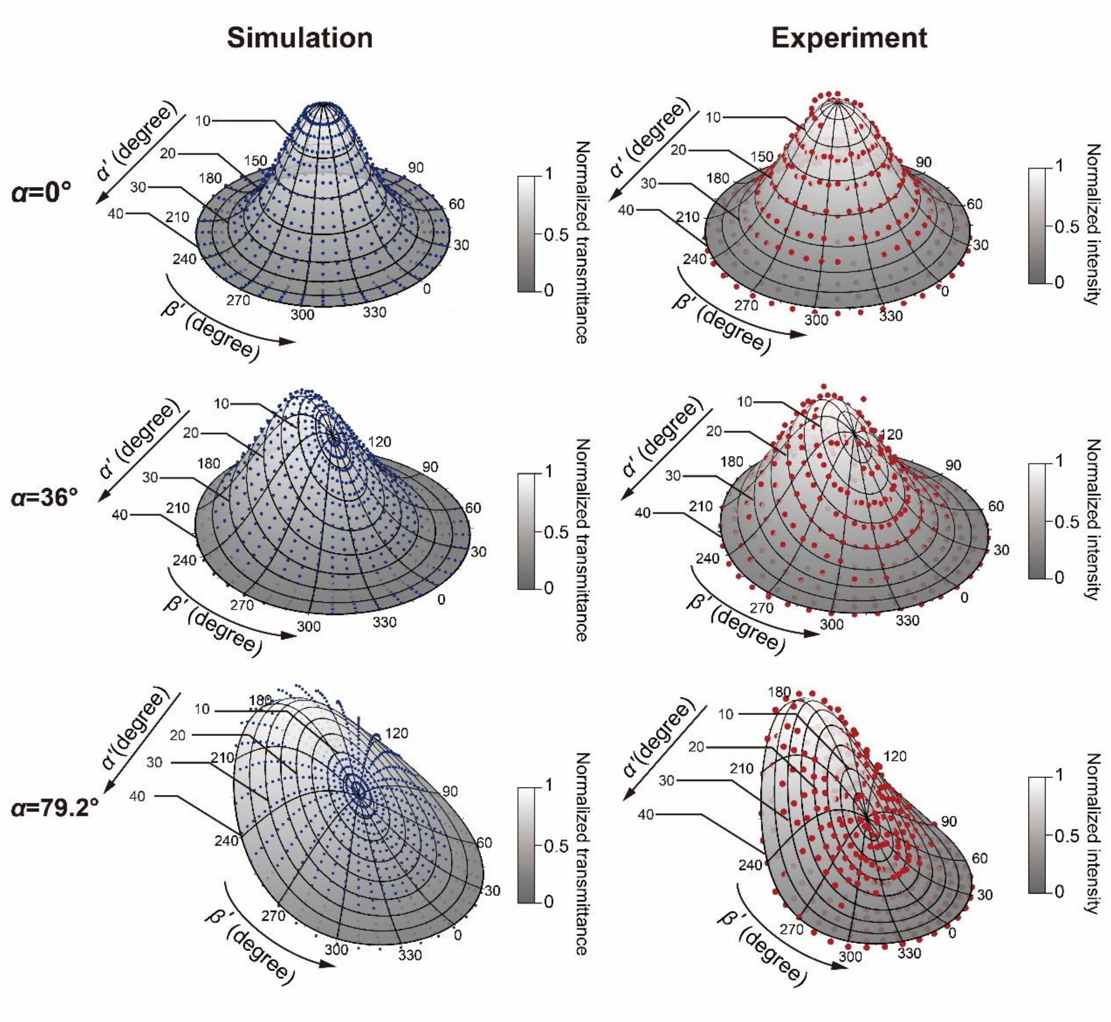

Supplementary Fig. 21. The angular sensitivity function of the ommatidia with orientations of (*α=0º, β=0º*), (*α=36º, β=0º*) and (*α=79.2º, β=0º*) respectively. Blue dots: the normalized transmittance obtained from the simulation. Red dots: the normalized intensity obtained from the experimental measurements. The grey surface: Gaussian-fitting surface.

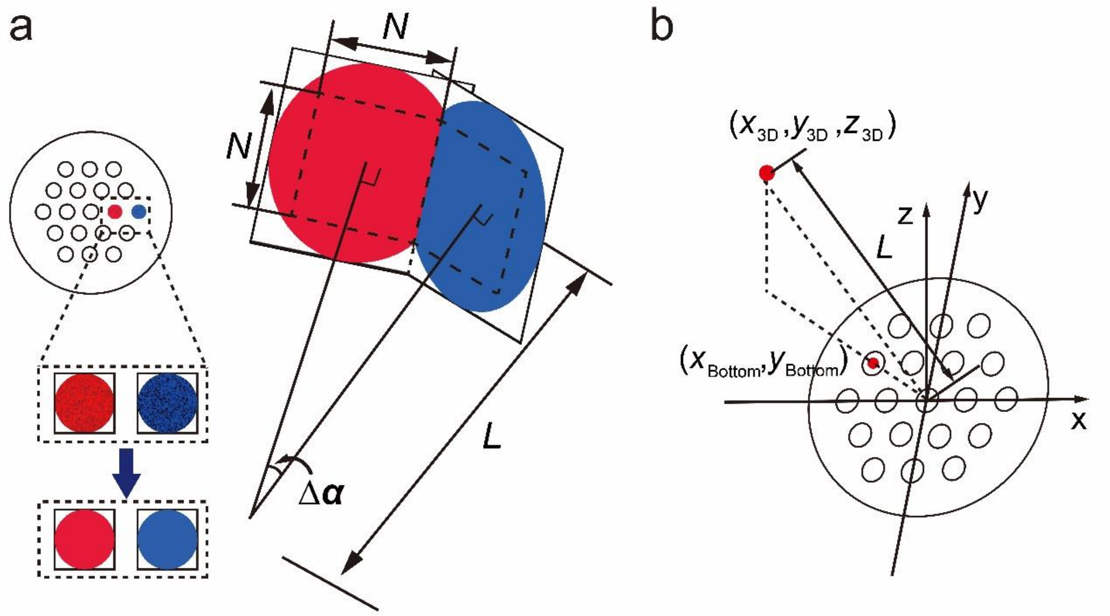

Supplementary Fig. 22. Reconstruction of a panoramic image. a, Schematic diagram of image stitching. **b,** The coordinate system used for the hemispherical imaging.

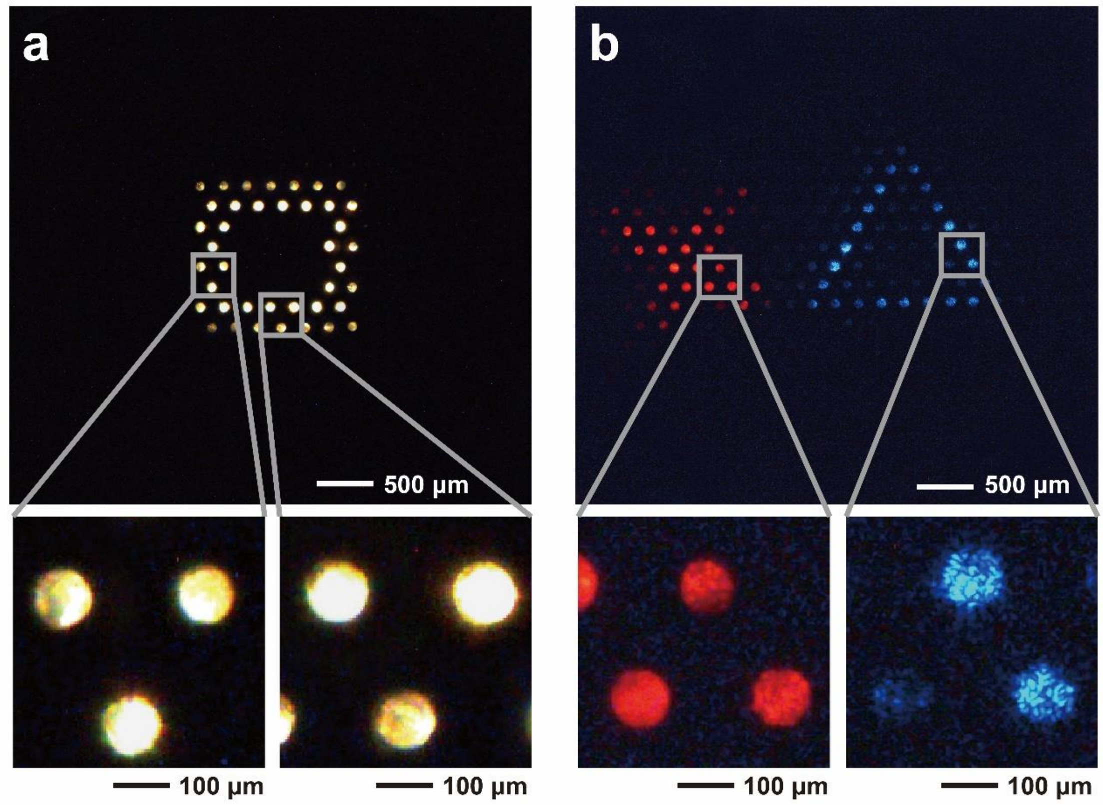

Supplementary Fig. 23. Images on the flat base of the compound eye that are detected by the camera. a, An image of a square placed in front of the compound eye. **b,** An image of a red cross fixed at an angular position of (*α=60º, β=0º*) and a blue triangle centred at (*α=20º, β=180º*).

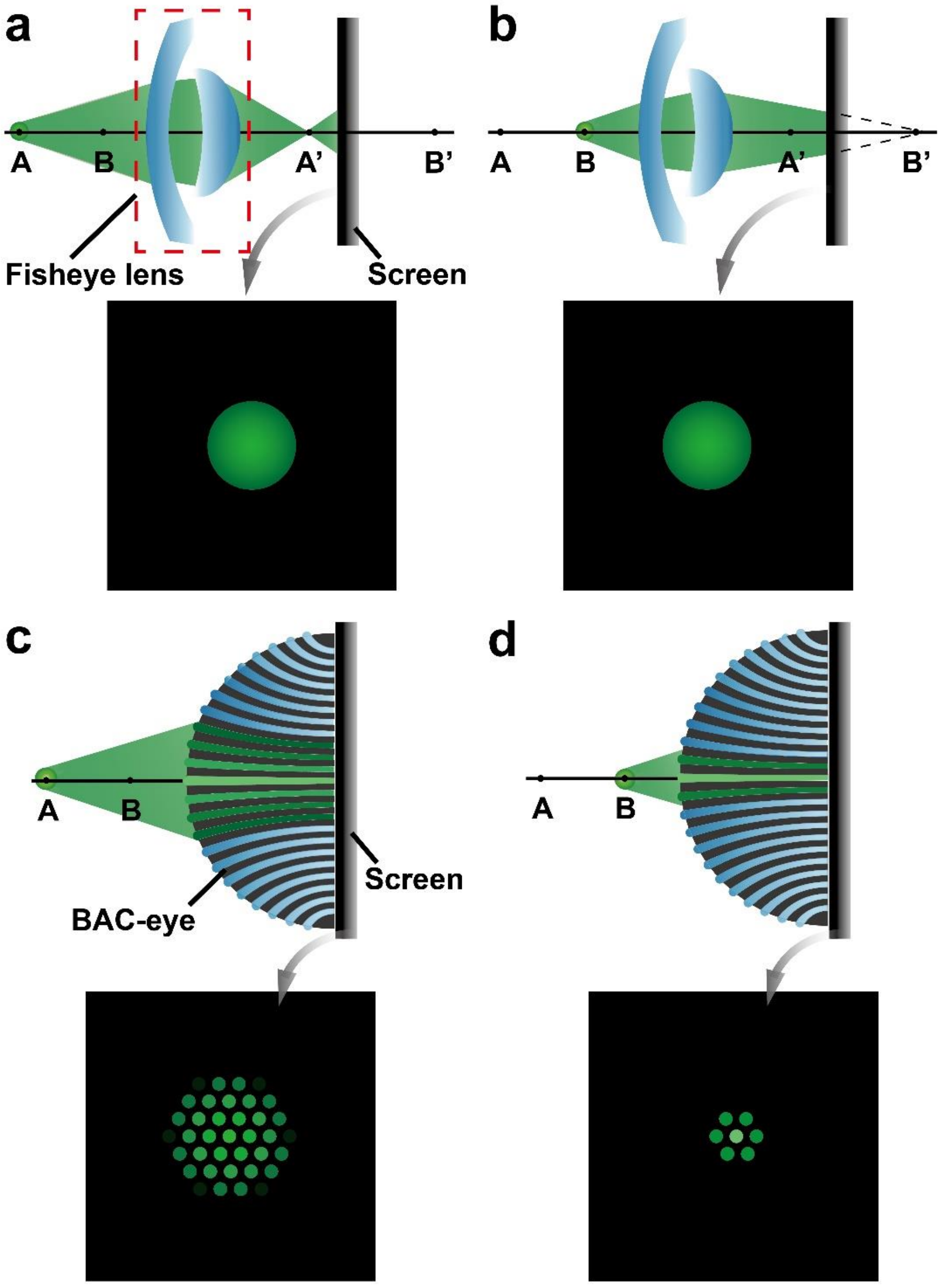

Supplementary Fig. 24. Schematic diagrams comparing the depth perception of a conventional fisheye lens and the BAC-eye. a, A point source at a far distance to the conventional lens and the image captured behind the lens. **b,** A point source at a close distance to the conventional lens and the image captured behind the lens. **c,** A point source at a far distance to the BAC-eye and the image captured at the flat base. **d,** A point source at a close distance to the BAC-eye and the image captured at the flat base.

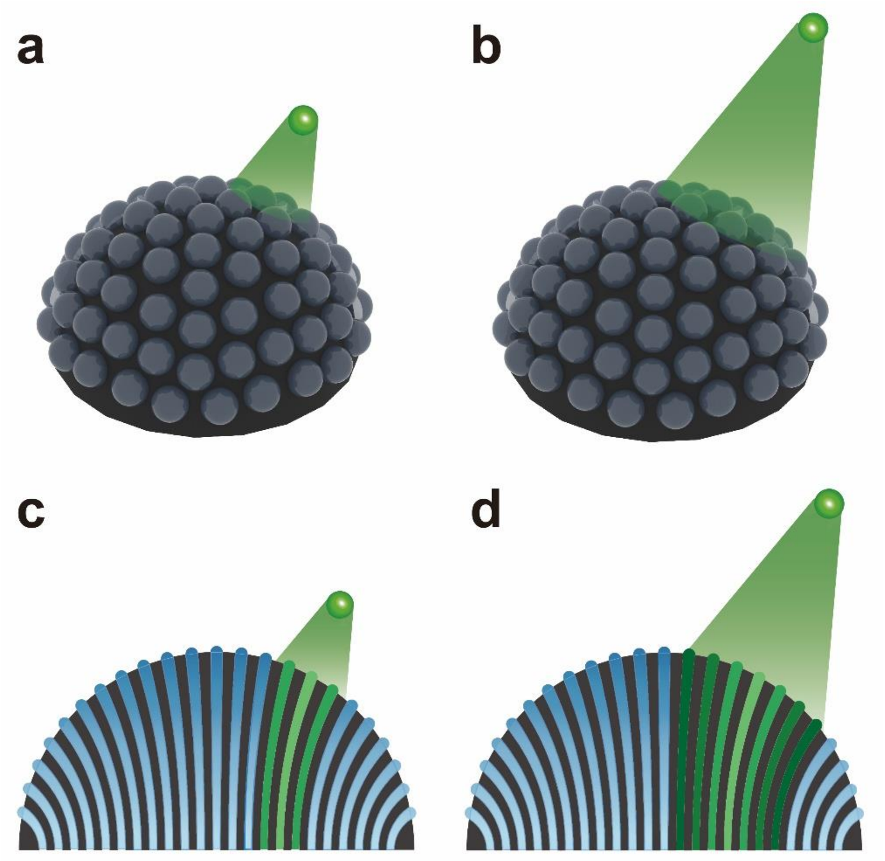

Supplementary Fig. 25. Schematic diagrams of the illumination of the point source on the compound eye. a, A point source at a close distance to the compound eye. **b,** A point source at a far distance to the compound eye. **c,** The cross section of the eye showing the light propagation inside the eye when the point source is close to the compound eye. Only a few ommatidia collect the light. **d,** The cross section of the eye showing the light propagation inside the eye when the point source is far away from the compound eye. More ommatidia collect the light from point sources that are farther away from the compound eye.

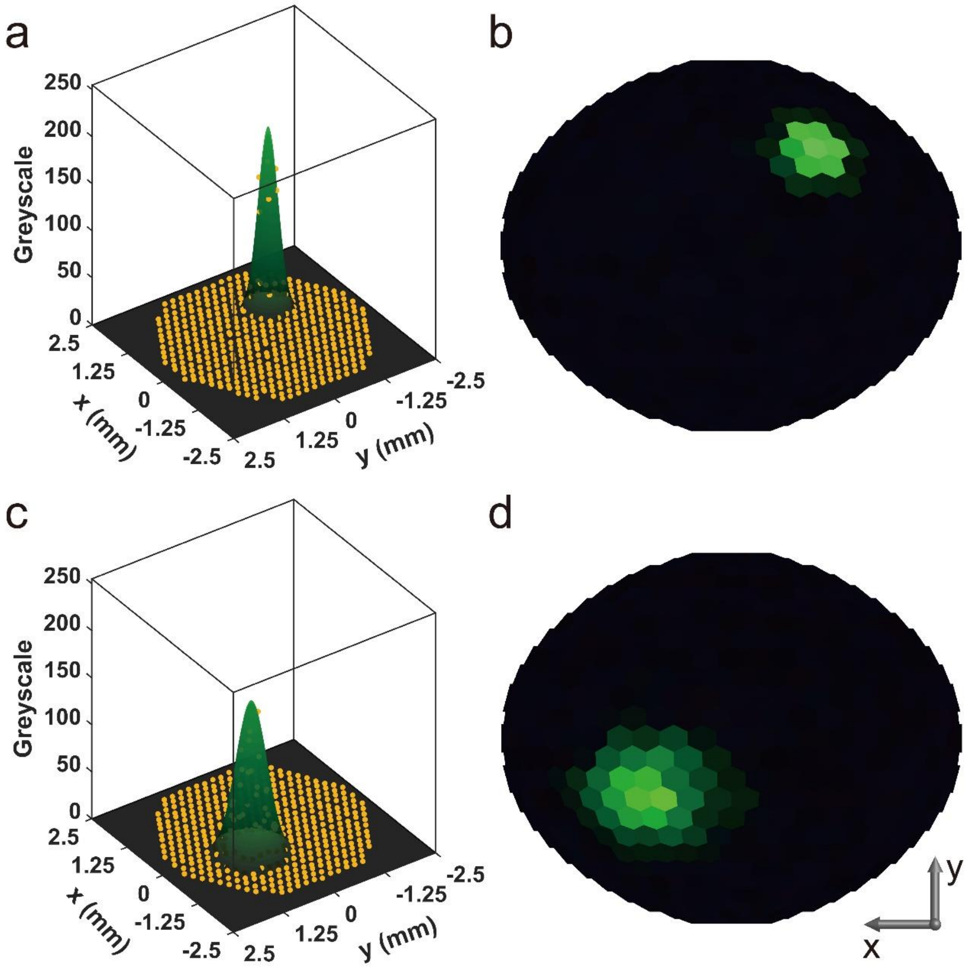

Supplementary Fig. 26. Positioning of the spotlight sources. a and c, The intensity distribution obtained by the BAC-eye and fitted with Gaussian functions. **b and d**, The reconstructed hemispherical images of the light spots. The target and measured positions of the spotlight source are marked in Fig. 5b.

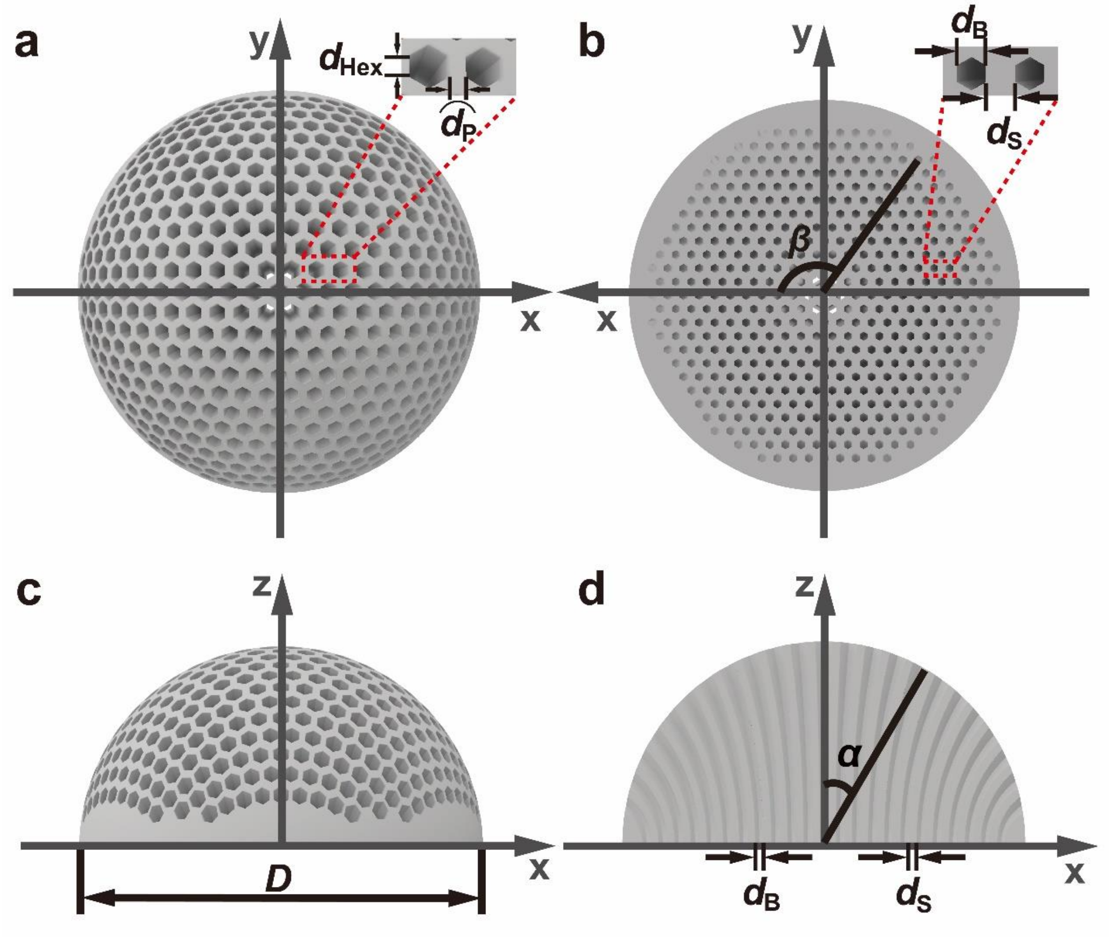

Supplementary Fig. 27. Design of the substrate for hexagonal microlenses and waveguides. a, Top view of the substrate. **b,** Bottom view of the substrate. c, Side view of the substrate. **d,** Cross section of the substrate.

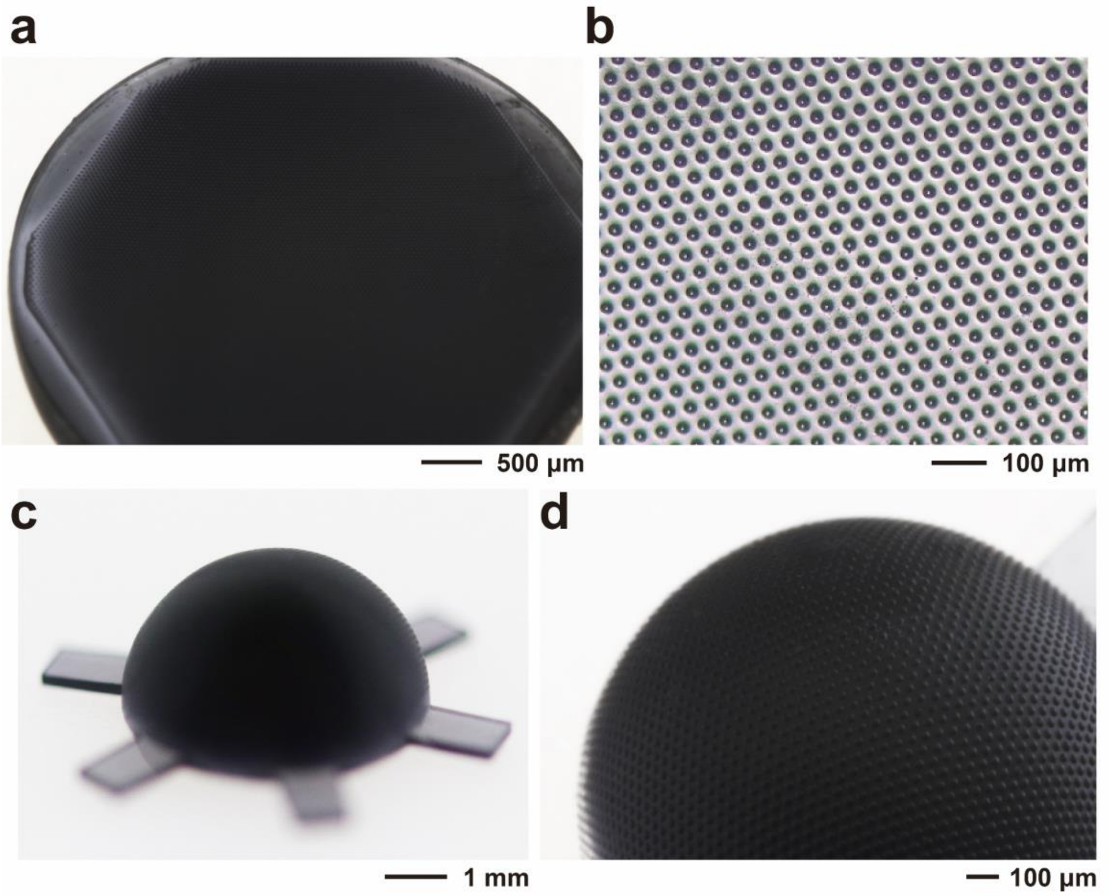

Supplementary Fig. 28. BAC-eye with 12,072 ommatidia. a, Base of the BAC-eye. **b,** A magnified image of the base. **c,** An image of the 3D printed component of the BAC-eye. **d,** A magnified image of the fabricated BAC-eye.

Supplementary Notes

Supplementary Note 1: Microfluidic-assisted moulding used in the BAC-eye fabrication

The BAC-eye consists of two main components: a single hemispherical substrate with silicone waveguides throughout it to guide the light to the image sensor, and a regular array of microlenses within posts which protrude from the surface of the hemisphere and collect the light. The structure of the microlenses and the cylindrical posts on the hemispherical surface of the BAC-eye are formed by microfluidic-assisted moulding *via* complementary replication of the mould structure shown in Fig. 1f of the main text. Since the lenses and posts are critical for properly collecting and transmitting light into the waveguides underneath, the formation of convex microlenses on the surface of the hemisphere is a critical step for a properly functioning BAC-eye.

The formation of the 3D microlens arrays is realized using a microfluidic-assisted moulding technique. First, a mould with an open hemispherical pit is 3D printed with microholes distributed across its surface. Then, the hemispherical pit is filled with acrylate resin. After that, the mould is spun at a spin rate of *Ω rpm*. The theoretical model of the mould formation is explained in the next section. When the rotational speed was larger than a critical value Ω_c=*1,385 rpm*, no acrylate resin remained within the main hemispherical pit of the mould, and all of the resin that remained was contained within the microholes. Therefore, spinning the mould above this threshold value ensured that resin only remained within the holes, where the curved fluid-air interface could then be cured to form the requisite lens surfaces.

The acrylate resin that remains in each cylindrical microhole is subject to gravity, a centrifugal force, and surface tension, and understanding how these forces contribute to the finalized geometry is critical to successful BAC-eye fabrication. The numerical simulations for the analysis of the acrylate resin in the microholes at different orientations (α) within a spinning mould and after the spinning are presented in Fig. 2b and Supplementary Fig. 5. The surface of the liquid-state acrylate resin in an on-axis microhole ($\alpha=0^o$) deforms symmetrically around the spinning axis and forms a parabolic shape. The surface of the liquid-state acrylate resin in the off-axis microholes ($\alpha\neq0^o$) shows a gradual tilt towards the outer part of the microhole as the angle α increases. The time duration for the spinning should be long enough to achieve mechanical equilibrium between gravity, centrifugal, and surface tension forces, under which condition no more acrylate resin is ejected from the microholes.

When the spinning stops, the mould is placed in a dark environment for 30 minutes. At this point, there is no centrifugal force and the effect of gravity on the liquid surface can be neglected because the diameter of the microhole is much smaller than the capillary length, $l_c = \sqrt{\gamma/(\rho g)}$, where γ is the acrylate resin-air surface tension ($\gamma=31\times10^{-3}$ *N/m*), ρ is the density of the acrylate resin

($\rho=1.02\times10^3$ kg/m^3), and g is the acceleration due to gravity ($g=9.8$ m/s^2). Thus, the surface tension plays a dominate role to deform the surface of the acrylate resin into a concave shape within the microholes. The curvature of the concave acrylate resin is the same regardless of the orientation of the microhole. In this regard, each microhole can be treated as an individual capillary tube, where the concave surface of the acrylate-resin fluid is determined by the interaction of the interfacial tension of the acrylate resin (liquid phase), the sidewall of the microhole (solid phase), and the air above the acrylate resin (gas phase). Therefore, the curvature radius of the concave surface can be expressed as $R = d/\left(2\cos(\theta_e)\right)$, where θ_e is the contact angle between the three phases under thermodynamic equilibrium. Once the desired concavity for microlens mould is achieved, the acrylate resin within the microholes is UV cured.

Consequently, the convex microlenses can be formed by placing the hemispherical eye into the mould, and curing a silicone elastomer into the shape of the lens and waveguides. The curvature of the convex lenses is identical to those of the concave surface of the acrylate resin within the microholes but have opposite sign (Fig. 2c-e).

We have demonstrated empirically and through numerical simulations that this microfluidic-assisted moulding process yields a desired convex microlens on top of cylindrical posts of various lengths. Each of these posts is connected to the bottom flat surface of the BAC-eye through a waveguide, using the same material and is hence perfectly optically matched. The diameter of each cylindrical post is slightly larger than that of the connecting waveguides to increase the contact area between the outer portion (the microlens/post system) and the internal structures (hemispherical substrate/optical pathways) for safely demoulding the substrate. This variation in diameter has a low influence on the efficiency of light coupling into the waveguide because the light is converged at the central area of the waveguide (Fig. 1b and Fig. 3i).

Supplementary Note 2: Theoretical model of the mould formation

We have developed a two-stage analytical model for the mould formation. The first stage is when the mould is spinning. The centrifugal force plays an important role, resulting in microholes that are partially filled with acrylate resin. In dynamic equilibrium, the surface of the acrylate resin remaining in a microhole with an orientation of *α* can be described as follows:

$$z_{\mathrm{Spin}\_\alpha}(\Omega) = \frac{\Omega^2(x^2+y^2)}{2g} - \frac{\Omega^2 D^2 \sin^2(|\alpha|+\varphi)}{8g} + \frac{1}{2}\sqrt{D^2-d^2} + \frac{d}{2}\cos(|\alpha|)\tan(\delta) + \frac{D}{2}(\cos(|\alpha|) - \cos(|\alpha|+\varphi) - 1) + H\cos(|\alpha|) - \sqrt{T_1+T_2+T_3} \quad (1)$$

in which

$$T_1 = \frac{\left(d\cos(|\alpha|)\tan(\delta) + 2H\cos(|\alpha|)\right)^2 - d^2\tan^2(\delta)}{4} + \frac{d^2}{4\cos^2(\delta)} - x^2 \quad (2)$$

$$T_2 = \left(y + \frac{D}{2}\sin(|\alpha|)\right)^2 + H^2 + dH\tan(\delta) \quad (3)$$

$$T_3 = \sin(|\alpha|)\left(y + \frac{D}{2}\sin(|\alpha|)\right)(d\tan(\delta) + 2H) \quad (4)$$

where *Ω* is the spin rate, *D* is the diameter of the hemispherical pit in the mould, *d* is the diameter of the microhole, *g* is gravitational acceleration, *H* is the depth of the microhole, $\varphi=\arcsin(d/D)$ and $\delta=\alpha-\arctan(\Omega^2 d/2g)+90^o$.

To avoid leaving any residual acrylate resin in the hemispherical pit, the rotational rate should be higher than a critical value, Ω_c, which has the form:

$$\Omega_c = \sqrt{\frac{32g}{3D}} \quad (5)$$

The rotational rate is inversely proportional to the amount of acrylate resin left in the microholes. At a very high spin speed, too little acrylate is left in the microholes, which makes it difficult to form a curved surface. Thus, the volume of the residual acrylate resin in any microhole, $V_{\mathrm{Spin}\_\alpha\_\mathrm{Residual}}$, should be no less than the volume, $V_{\mathrm{Static\_min}}$, which is sufficient to form a continuous concave surface in the microhole after spinning, *i.e.* $V_{\mathrm{Spin}\_\alpha\_\mathrm{Residual}} \geq V_{\mathrm{Static\_min}}$.

$$V_{\mathrm{Spin}\_\alpha\_\mathrm{Residual}}(\Omega) = \int_{-d/2}^{d/2}\int_{-d/2}^{d/2} Z_{\mathrm{Spin}\_\alpha}(\Omega)\,dX\,dY \quad (6)$$

and

$$V_{\mathrm{Static\_min}} = \frac{\pi d^3}{8}\left(\frac{90^{\circ}-\theta_e}{135^{\circ}\times\cos^3(\theta_e)} - \frac{\tan(\theta_e)}{3}\right) \quad (7)$$

where

$$\begin{bmatrix} X \\ Y \\ Z_{\mathrm{Spin}\_\alpha}(\Omega) \end{bmatrix} = \begin{bmatrix} 1 & 0 & 0 \\ 0 & \cos(\alpha) & \sin(\alpha) \\ 0 & -\sin(\alpha) & \cos(\alpha) \end{bmatrix} \begin{bmatrix} x \\ y \\ z_{\mathrm{Spin}\_\alpha}(\Omega) \end{bmatrix} \quad (8)$$

and θ_e is the contact angle between the acrylate resin, cured photosensitive polymer and air under a thermodynamic equilibrium. The volume of the residual

acrylate resin in the microholes can be numerically calculated for any given spin rate. Thus, the maximum spin rate can be calculated.

The second stage of this model is after spinning. Each microhole acts as a capillary tube. The capillary effect acts to curve the surface of the residual acrylate resin in the microhole. The curvature of the acrylate resin surface is:

$$R = \frac{d}{2\cos(\theta_e)} \quad (9)$$

Above this concave surface, there is a section of cylindrical vacancy due to the loss of the acrylate resin during spinning. The depth of this vacant cylinder, *i.e.* the height of the post under the microlens, is

$$h_{\mathrm{Post}} = H - \frac{4V_{\mathrm{Spin\_}\alpha\mathrm{\_residual}}}{\pi d^2} - \frac{d}{2}\left(\frac{90^{\circ} - \theta_e}{135^{\circ} \times \cos^3(\theta_e)} - \frac{\tan(\theta_e)}{3}\right) \quad (10)$$

In static equilibrium, the location of the surface of the acrylate resin becomes

$$z_{\mathrm{Static\_}\alpha}(\Omega) = \sqrt{\frac{d^2}{4\cos^2(\theta_e)} - x^2 - \left(y - \sin(\alpha)\left(\frac{D}{2}\cos(\varphi) + h_{\mathrm{Post\_}\alpha} + d\tan(\theta_e) - \frac{d}{\cos(\theta_e)}\right)\right)^2} + \frac{D}{2} + H - \frac{d}{2}\tan(\varphi) - \cos(\alpha)\left(\frac{D}{2}\cos(\varphi) + h_{\mathrm{Post\_}\alpha} + d\tan(\theta_e) - \frac{d}{\cos(\theta_e)}\right) \quad (11)$$

In the design of the BAC-eye, the diameter of the microholes in the mould should be less than the capillary length, *i.e.* $l_c = \sqrt{\gamma/(\rho g)}$, where γ is the surface tension at the acrylate resin-air interface, and ρ is the density of the acrylate resin. Thus, the resulting structure of the microlens array and the posts can be predicted based on this model. In order to realize relatively uniform posts, a spin rate which is slightly higher than the critical spin rate is used in practice.

Supplementary Note 3: Design of the hemispherical substrate of the BAC-eye

Supplementary Fig. 3 depicts the design procedure for the hemispherical substrate. The size of the BAC-eye, *D*, the diameter of the waveguide proximal ends, d_B, and the distribution pattern of the ommatidia are first determined. The separation distance, d_S, between the two proximal ends of the adjacent waveguides on the flat base is set to the minimum dimension that the 3D printer is able to manufacture. The number of ommatidia in the compound eye with a diameter of *D* can be expressed as

$$M = 3m^2 + 3m + 6(m - 3) \tag{12}$$

where

$$m = \left\lfloor \frac{D - d_{\mathrm{B}}}{2(d_{\mathrm{B}} + d_{\mathrm{S}})} \right\rfloor \tag{13}$$

$\lfloor\cdot\rfloor$ is the greatest integer function.

Then, the distribution of waveguide exits at the flat bottom of the hemisphere need to be arranged. After that, the routing of hollow pipelines within the hemisphere is performed using the following design rules.

An auxiliary axis, labelled as the *w*-axis, passes through the origin point of the hemisphere and the centre of a waveguide end (on the flat bottom) for a specific ommatidium. This yields a cross section of the hemisphere defined by the *wz*-plane. In this cross section, two edges of the waveguide end (as it exits the flat bottom of the substrate) are marked as *A* and *A'*. On the hemisphere, two edges of an opening hole, marked as *B* and *B'*, are located where *OB* and *OB'* cut the hemisphere with angles of *θ* and *θ'* with respect to the z-axis. The angles can be calculated as follows.

$$\theta = \frac{2L_{\mathrm{OA}}}{D} \times 90° \tag{14}$$

$$\theta' = \frac{2L_{\mathrm{OA'}}}{D} \times 90° \tag{15}$$

where L_{OA} and $L_{OA'}$ are the distance from the origin point to point *A* and point *A'*, respectively. Afterwards, two perpendicular bisectors of the lines *AB* and *A'B'* are plotted as auxiliary lines *CD* and *C'D'*. The points *D* and *D'* are the points where *CD* and *C'D'* intersect with the *w*-axis. The boundaries of the pipelines, *i.e.* the curves $\overset{\frown}{AB}$ and $\overset{\frown}{A'B'}$, are determined with *D* and *D'* as the centre points and *AD* and *A'D'* as radii, respectively. Consequently, all the remaining pipelines can be designed by repeating the above steps. Hence, the direction of the opening of the pipeline on the hemisphere surface actually determines the angular orientation of the ommatidium. The polar angle, *α*, is *(θ+θ')/2* and the azimuthal angle, *β*, is the angle of intersection between the *x*-axis and *w*-axis.

Supplementary Note 4: Optical density of the photosensitive polymer dyed with Sudan Black 3 solvent dye

A substrate comprised of photosensitive polymer was used to support and isolate the ommatidia. The photosensitive polymer was dyed with Sudan Black 3 solvent dye (Sigma-Aldrich, USA) to absorb the light that escapes from the waveguides. Supplementary Fig. 8a shows a slice of the photosensitive polymer before adding the solvent dye. Supplementary Figs. 8b-d show slices of the photosensitive polymer mixed with solvent dye at concentrations of 900 μg/mL, 1200 μg/mL, and 1500 μg/mL, respectively. The polymer, which is usually yellow without the solvent dye, transitions to black as the concentration of the dye increases. Each slice shown in the figures has a thickness of 100 μm to mimic the smallest separation distance between two adjacent waveguides within the BAC-eye. Supplementary Fig. 8e shows the optical density of the polymer over the entire visible spectrum as the concentration of the dye increases. When the concentration is 1,500 μg/mL, the material, which has a high optical density of 3.23, can efficiently absorb the light that leaks out of the waveguides.

Furthermore, the optical density of the thin photosensitive polymer film dyed with the solvent dye at a concentration of 1500 μg/mL was evaluated. The photosensitive polymer was spin coated on a glass substrate and UV cured. Half of the film was then peeled off from the glass to measure the optical density (Supplementary Fig. 9). The height of the film remaining on the glass substrate was also measured. The thickness of the film was 9.8 μm and the optical density over the entire visible spectrum was larger than 3.

Supplementary Note 5: Optical loss in the ommatidia

The experimental setup for measuring the optical loss in the ommatidia is depicted in Supplementary Fig. 17. A tungsten-halogen light source (SLS201L/M, Thorlabs, Inc., USA) was used. The light output from a multi-mode fibre (M67L02, Thorlabs, Inc., USA) was normally incident on the microlens of the ommatidium. The distance between the fibre tip and the ommatidium was 2 mm. The light output from the flat bottom of the waveguide was collected by an aspherical lens (C151TMD-A, Thorlabs, Inc., USA). The optical power was then measured by a photodiode power sensor (S130VC, Thorlabs, Inc., USA). The incident power was measured directly from the tip of the multi-mode fibre by placing the aspherical lens and the power sensor at the tip of the fibre. The loss, including the coupling loss into/out of the ommatidium and the transmission loss in the waveguide, can be calculated as follows.

$$TL = 10\log_{10}\frac{I_{\mathrm{in}}}{I_{\mathrm{out}}} \tag{16}$$

where I_{in} is the incident optical power and I_{out} is the output optical power. In our measurement, the incident power was 42 mW. The output power measured for the ommatidia with orientations of *(α=0º, β=0º)*, *(α=36º, β=0º)* and *(α=79.2º, β=0º)* was 19.3 mW, 14.3 mW and 12.2 mW, respectively. The loss in the ommatidia was 3.38 dB, 4.68 dB and 5.37 dB, respectively.

Supplementary Note 6: Angular sensitivity of the ommatidia

The simulation for investigating angular sensitivity of the ommatidia was performed by using the ray tracing module in COMSOL Multiphysics ® software. A model for individual ommatidium was established, and the structures of each ommatidium such as the curvature of the microlens, the height of the post and the degree of curvature of the waveguide exactly follow those in the design and the experiment. The physical properties, including the refractive indices and optical density of the materials were the same as those obtained in the experimental measurement. The collimated light was incident into the ommatidia at different incident angles (polar angle *α'* and azimuthal angle *β'*, as defined in Supplementary Fig. 13). The transmittance, *i.e.* the ratio between the output power and the input power, was calculated. Supplementary Fig. 21 illustrates the calculated angular sensitivity function for the ommatidia with orientations of *(α=0º, β=0º)*, *(α=36º, β=0º)* and *(α=79.2º, β=0º)* obtained by the simulation. The transmittance was sensitive to the incident angles. The angular sensitivity function revealed that the central ommatidium (*α=0º*) can receive most of the light when the incident light is normal (along *z'*-axis) to the microlens, while the other ommatidia can achieve the highest transmittance when the incidence is oblique to *z*-axis (as defined in Supplementary Fig. 13).

In addition to the simulations, experiments were also conducted to compare the observed angular sensitivity with the simulation results. The experimental setup for measuring the angular sensitivity of the ommatidia is illustrated in Supplementary Fig. 21. The collimated light from a broadband incoherent light source (SLS201L/M, Thorlabs, Inc., USA) via a fibre collimator (F810FC-543, Thorlabs, Inc., USA) was incident on the ommatidium with different incident angles (*α'=0º* to *40º* and *β'=0º* to *350º*). Behind the compound eye, a CMOS camera (EO-18112, Edmund Optics Inc., USA) mounted with a telecentric lens (1X, 40mm WD CompactTL™, Edmund Optics Inc., USA) was used to capture the image on the flat base of the eye. An average gray-scale value representing the output intensity of the ommatidium was calculated based on the 80×80 pixels in the sub-image from the corresponding waveguide. All of the values for the different ommatidia and the different incident angles were normalized with respect to the maximum value measured at the central ommatidium (*α=0º*). The normalized values are denoted by I_{Norm}. The experimental data for each ommatidium were fitted with a Gaussian function as follows.

$$I_{\text{Norm}}(x,y) = p_1\left(\mathrm{e}^{-\left(\frac{x-p_2}{2p_4}\right)^2} + \mathrm{e}^{-\left(\frac{y-p_3}{2p_5}\right)^2}\right) \tag{17}$$

where

$$x = \alpha'\cos(\beta') \tag{18}$$

$$y = \alpha'\sin(\beta') \tag{19}$$

and p_1, p_2, p_3, p_4 and p_5 are the parameters to be determined in the fitting.

Supplementary Fig. 21 shows the measured angular sensitivity function. The red dots are the normalized intensity obtained from experimental

measurements and the surface was obtained using Gaussian fitting. The experimental results agree well with the simulation results. In the central ommatidium ($\alpha=0^o$), the maximum intensity was obtained when the light was normally incident on the ommaitdium, *i.e.*, ($\alpha'=0^o$, $\beta'=0^o$). The ommatidia with orientations of ($\alpha=36^o$, $\beta=0^o$) and ($\alpha=79.2^o$, $\beta=0^o$) have the maximum output at incident angles of ($\alpha'=12^o$, $\beta'=180^o$) and ($\alpha'=30^o$, $\beta'=180^o$). The light incident into the ommatidia from these angles does not suffer any abrupt changes in direction of propagation and can efficiently propagate along the curved waveguide.

Supplementary Note 7: Optimization of the BAC-eye

The number of the ommatidia and the filling factor of the BAC-eye are key parameters affecting the imaging performance, *i.e.*, the resolution and the sensitivity. The BAC-eye can be further optimized according to the strategies as follows.

The number of the ommatidia is one of the most important criteria of the compound eye. A compound eye with a large number of ommatidia can achieve wide-angle panoramic imaging with a high resolution and a high density of pixels. In the BAC-eye, each ommatidium is independent and isolated by the substrate. Even with a high number of ommatidia at small separation distances, they do not interlace with one another. Thus, the number of ommatidia used in the BAC-eye are not limited structurally. The minimum feature size that a 3D printer is able to manufacture is a limiting factor for device fabrication, which determines the minimum dimension between ommatidia in the substrate. In the BAC-eye, the separation between the adjacent waveguides on the flat base of the substrate, d_S, is set as the minimum dimension because it is inversely proportional to the number of the ommatidia. If a 3D printer with a higher printing resolution is used, the separation distance between the waveguides can become narrower and the number of the ommatidia could be significantly increased. It is worth noting that the reduction of the separation distance would not cause severe optical crosstalk because of the high optical density of the substrate.

The filling factor is another important criterion for improving the imaging performance of the BAC-eye. A compound eye with a high filling factor has a high resolution and high sensitivity for imaging. The filling factor is related to the aperture size of the microlenses and the separation between the microlenses. The aperture size of the microlenses can be enlarged by modifying the shape of the microlenses and the cross-section of the waveguides into a hexagon, which is an efficient shape for a close-packed structure. In addition, the separation between the microlenses can be reduced to the minimum feature size that a 3D printer can produce. In the design, the parameters, θ and θ', which determine the opening hole for the waveguide, $\overset{\frown}{BB'}$ as defined in Supplementary S3, should be modified as follows.

$$\theta = \frac{1}{D}\left((2L_{\mathrm{OA}} + d_{\mathrm{B}}) \times 90° - \overset{\frown}{\mathrm{d}_{\mathrm{P}}}\right) \quad (20)$$

$$\theta' = \frac{1}{D}\left((2L_{\mathrm{OA'}} - d_{\mathrm{B}}) \times 90° + \overset{\frown}{\mathrm{d}_{\mathrm{P}}}\right) \quad (21)$$

where $\overset{\frown}{d_P}$ is the minimum feature size that a 3D printer can manufacture and its value can be set to d_S.

Supplementary Fig. 27 illustrates the modified design of the substrate for

the hexagonal microlenses and waveguides. The side of the hexagon is 100 μm and the separation is 100 μm, which are based on the minimum feature size of the 3D printer (nanoArch® P140, BMF Precision Technology Co., China) used in the experiment. The optimized design can enlarge the aperture of the microlenses by 106.4%.

Furthermore, the number of ommatidia can be significantly increased by using a 3D printer with a higher resolution. For example, the nanoArch® S130 (BMF Precision Technology Co.) has a printing resolution of 2 μm, a minimum layer thickness of 5 μm, and a maximum build size of 50 mm × 50 mm × 10 mm. Supplementary Fig. 28 shows a BAC-eye that was fabricated with this printer, containing a total of 12,072 ommatidia. The mould was prepared by spinning the acrylate resin in the hemispherical pit with a speed of 1,000 rpm. The diameter of this BAC-eye is also 5 mm. Both the separation of the adjacent waveguides (d_S) and the diameter of the waveguide (d_B) on the flat base of the eye are 20 μm.
